# Emerging Technologies in RNA–Protein Interaction Analysis

**DOI:** 10.3390/biology15090680

**Published:** 2026-04-26

**Authors:** Nishinki T. Muthumuni, Jia Guo

**Affiliations:** 1 Biodesign Institute, Arizona State University, Tempe, AZ 85287, USA; mndesilv@asu.edu; 2 School of Molecular Sciences, Arizona State University, Tempe, AZ 85287, USA

**Keywords:** RNA–protein interactions, RNA-binding proteins, CLIP-seq, proximity labeling, imaging-based approaches, CRISPR/Cas13, RNA interactome

## Abstract

RNA–protein interactions (RPIs), mediated primarily by RNA-binding proteins (RBPs), are central to post-transcriptional gene regulation and govern RNA splicing, transport, localization, translation, and decay. Dysregulation of RBPs and their associated RNA networks contributes to diverse pathologies, including cancer, neurodegenerative disorders, and viral infections. However, profiling RPIs remains a challenge due to their inherent transience, low binding affinity, and shifting spatial dynamics. This review provides a comprehensive and systematic overview of current methodologies for investigating RPIs. We discuss RNA-centric and protein-centric strategies. In addition, imaging-based approaches are evaluated for their capacity to resolve spatial and temporal dynamics of RBP–RNA interactions in situ. We compare these methodologies in terms of resolution, sensitivity, specificity, and biological applicability, emphasizing the importance of integrative strategies for constructing high-resolution, context-dependent RPI maps in physiological and disease settings.

## Simple Summary

Cells rely on interactions between RNA molecules and proteins to control how genes are used, which affects processes such as growth, development, and response to disease. However, studying these interactions is challenging because they are often weak, short-lived, and occur in specific locations inside cells. This review explains recent advances in technologies that allow scientists to better detect and understand these interactions. We describe methods that focus either on the RNA or the protein, as well as imaging approaches that allow visualization of these interactions inside cells. Each method has strengths and limitations, such as differences in sensitivity, specificity, and ability to capture dynamic changes. No single technique can provide a complete picture, so combining multiple approaches is often necessary. These advances are helping researchers build more detailed maps of how RNA and proteins work together in both healthy and diseased cells. A better understanding of these processes can improve our knowledge of conditions such as cancer, brain disorders, and viral infections, and may ultimately support the development of new diagnostic tools and treatments.

## 1. Introduction

### 1.1. The Biological Significance of RNA–Protein Interactions

RPIs constitute a central regulatory layer in cellular biology and are essential for virtually all stages of RNA metabolism, including transcriptional control, RNA maturation, intracellular transport, and protein synthesis [1]. Through these interactions, RNA molecules acquire specific functional identities, as RPIs determine RNA stability, localization, and translational efficiency, thereby shaping post-transcriptional gene regulation. RBPs are the primary mediators of these processes, recognizing defined RNA sequence motifs or structural elements within regions such as the 5′ cap, 3′ untranslated regions (UTRs), coding sequences, and introns to modulate splicing, export, decay, and translation [2,3]. In addition to messenger RNAs, non-coding RNAs including long non-coding RNAs (lncRNAs) and small regulatory RNAs exert their biological functions largely through dynamic interactions with RBPs, contributing to chromatin organization, transcriptional regulation, and RNA interference pathways [4,5].

Dissecting the molecular principles governing RBP–RNA recognition and binding specificity is therefore critical for understanding both normal cellular physiology and disease pathogenesis. Aberrant RPIs have been strongly implicated in a wide range of human disorders, including neurodegenerative diseases such as Alzheimer’s disease (AD) and amyotrophic lateral sclerosis (ALS), multiple cancer types, and viral infections, where disruption of RBP function leads to widespread defects in RNA processing, transport, and translation [6,7,8]. Consequently, RBPs and their associated RNA networks have emerged as key determinants of cellular homeostasis and promising targets for therapeutic intervention.

### 1.2. Challenges in Studying RPIs

Investigating RPIs remains technically challenging due to their dynamic, transient, and context-dependent nature. Many RBP–RNA associations are weak or short-lived, making them difficult to capture using conventional biochemical approaches that rely on stable binding equilibria [9,10].

Additional complexity arises from the intracellular environment, where RNA and protein populations are continuously synthesized, modified, and degraded. Molecular crowding, competing interactions, and cofactor dependencies can significantly influence RBP–RNA association kinetics, complicating the isolation of intact complexes without disrupting physiologically relevant interactions [11,12].

RPIs are also highly spatially organized, occurring within distinct subcellular compartments such as the nucleus, cytoplasm, and membraneless organelles (e.g., stress granules and P-bodies). This spatial heterogeneity is tightly linked to function, requiring methods that integrate molecular specificity with spatial and temporal resolution [13,14].

Furthermore, RBPs often participate in multiple regulatory pathways, including splicing, transport, translation, and RNA decay, making it difficult to interpret interactions in isolation. This functional overlap necessitates integrative approaches to accurately define RPI networks within complex cellular systems [9,12,15].

Classical methods such as co-immunoprecipitation (Co-IP) and in vitro binding assays provide valuable insights but are limited by antibody dependence, low sensitivity for transient interactions, and lack of physiological context [16,17,18].

Collectively, these challenges highlight the need for advanced methodologies capable of capturing RPIs with improved sensitivity, resolution, and biological relevance.

### 1.3. Importance of Advanced Techniques in RPI Studies

Recent technological advances in next-generation sequencing (NGS) and single molecule imaging approaches have markedly expanded the scope and resolution at which RPIs can be interrogated [19]. These innovations enable the detection of RBP–RNA associations with high sensitivity across diverse cellular states and biological contexts. Widely used approaches such as crosslinking immunoprecipitation (CLIP) [20], RNA immunoprecipitation (RIP) [21], and RNA–proximity ligation assays (RNA–PLA) [22] have substantially improved our ability to profile protein–RNA associations involving both coding and non-coding RNAs. In addition to identifying interacting partners, these methodologies facilitate transcriptome-wide mapping of RBP binding sites and the characterization of RNA-binding domains within proteins [20].

Further refinements of these strategies have enabled increasingly precise delineation of RPI landscapes. For example, individual-nucleotide resolution CLIP (iCLIP) allows the identification of protein–RNA contact sites with single-nucleotide accuracy, providing detailed insights into binding specificity and regulatory mechanisms [23]. Proximity-labeling-based approaches such as APEX-Seq extend RPI analysis to living cells, capturing spatially restricted RNA–protein associations that are difficult to resolve using conventional biochemical methods [24]. Similarly, RPI detection by biotinylation (RaPID) has emerged as a robust platform for identifying dynamic and transient RPIs within native cellular environments [25]. In parallel, advances in liquid chromatography–tandem mass spectrometry (LC-MS/MS) have enabled large-scale, high-throughput identification of RNA-associated proteins, further expanding the capacity to reconstruct complex RPI networks [26]. Together, these methodologies complement classical approaches and provide a more comprehensive framework for mapping RPIs across diverse biological systems.

Despite these advances, no single method can fully capture the multifaceted nature of RPIs. High-throughput sequencing-based techniques excel at generating global interaction maps but often lack spatial resolution and may not fully capture the temporal dynamics of RNA–protein associations. In contrast, imaging-based approaches provide detailed spatial information and enable visualization of RPIs within specific cellular compartments, yet they may have limited sensitivity for weak or transient interactions. Consequently, the choice of experimental strategy must be guided by the specific biological question, with careful consideration of the required scale, spatial precision, and temporal resolution. Integrative use of complementary techniques is therefore essential for achieving a more complete and physiologically relevant understanding of RPI dynamics.

### 1.4. Objective of This Review

This review provides a systematic overview of current methodologies used to study RPIs, with an emphasis on their mechanistic principles, applications, and methodological limitations. Classical biochemical approaches, including CLIP [20] and RIP [21], are evaluated alongside emerging image-based and proximity-labeling techniques such as RNA–PLA [22] and RaPID [25]. These methods are compared in terms of sensitivity, spatial resolution, and their ability to capture transient and low-affinity interactions.

Recent advances in single-cell RPI profiling are also discussed, highlighting their capacity to resolve cell-to-cell heterogeneity and context-dependent RPI dynamics that are not accessible through bulk analyses [27,28]. Collectively, these technological developments offer new opportunities to interrogate RNA regulatory mechanisms with increased precision and biological relevance, thereby advancing understanding of RNA-mediated processes in both normal physiology and disease states, including neurodegeneration, cancer, and viral infection [6,8].

## 2. Methods for Detecting RNA–Protein Interactions

### 2.1. RNA-Centric Methods

RNA-centric methodologies place RNA molecules rather than proteins at the center of experimental design, enabling direct interrogation of RNA-associated molecular networks. These approaches treat RNA as an active regulatory scaffold and are particularly useful for defining RNA-associated protein landscapes in a spatial and temporal context. By capturing ribonucleoprotein (RNP) assemblies in native or near-native environments, RNA-centric approaches reveal layers of RNA-mediated regulation that are often obscured in protein-centric analyses [29,30].

**Figure 1:**
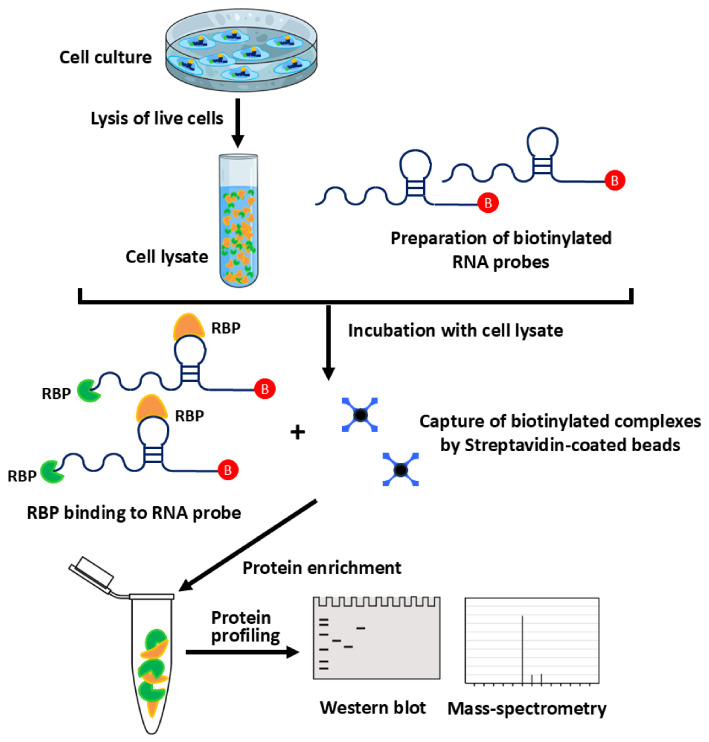
Schematic illustration of in vitro RNA-centric approaches. Biotinylated synthetic RNA probes are prepared and incubated with cell lysates containing RBPs (represented by orange and green icons). RBPs with affinity for the RNA sequence bind directly to the synthetic RNA probes. The resulting probe–protein complexes are captured using streptavidin-coated beads via the biotin tag, followed by washing to remove unbound components. Enriched RNA-binding proteins are subsequently analyzed by downstream methods such as immunoblotting or mass spectrometry.

**Figure 2:**
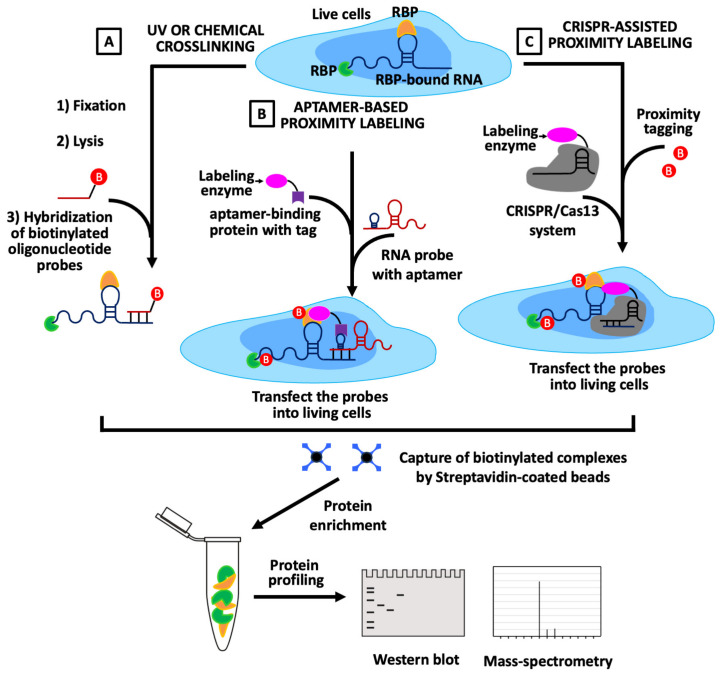
Schematic illustration of in vivo RNA-centric approaches. RBPs are represented by green and orange icons, and labeling enzymes are represented by pink icons. (**A**) In crosslinking-based approaches, RNA–protein complexes formed in living cells are stabilized by UV or chemical (e.g., formaldehyde) crosslinking and subsequently captured using biotinylated oligonucleotide probes after cell lysis. (**B**) In aptamer-assisted proximity labeling-based approaches, RNA molecules are targeted using aptamer-based systems that recruit aptamer-binding proteins fused to labeling enzymes, enabling biotinylation of proteins in close spatial proximity to the target RNA within living cells. While certain implementations such as HyPro-MS involve fixation and permeabilization, many proximity labeling strategies are performed in living cells. (**C**) In CRISPR-assisted proximity labeling approaches, catalytically inactive CRISPR/Cas13 (dCas13) systems are introduced into living cells to guide labeling enzymes to specific RNA targets, enabling proximity-dependent tagging of RNA-associated proteins. Finally, biotinylated RNA-associated proteins or RNA–protein complexes are enriched using streptavidin-coated beads and analyzed by downstream methods such as immunoblotting or mass spectrometry.

Current RNA-centric technologies can be broadly organized according to both methodological principles and experimental contexts, including in vitro reconstitution strategies (Figure 1) as well as in vivo approaches that preserve cellular architecture (Figure 2A–C). Figure 1 illustrates in vitro RNA-centric approaches based on probe-mediated pulldown of RNA–protein complexes, whereas Figure 2 summarizes in vivo strategies including crosslinking (Figure 2A), proximity labeling (Figure 2B), and clustered regularly interspaced short palindromic repeats (CRISPR)-assisted methods (Figure 2C). In vitro techniques, such as biotinylated RNA pulldown and aptamer-based affinity capture, use synthetic nucleic acid probes incubated with cell lysates to isolate associated proteins under controlled conditions. These approaches enable detailed examination of binding specificity and sequence or structural requirements for RPIs, but often lack native RNA modifications and cellular context.

In contrast, in vivo hybridization-based pulldown platforms including Chromatin Isolation by RNA Purification (ChIRP) [31], Capture Hybridization Analysis of RNA Targets (CHART) [32,33], RNA Antisense Purification followed by Mass Spectrometry (RAP-MS) [34], identification of Direct RNA Interacting Proteins (iDRiP) [35,36] and Multiple Oligo-Assisted RNA Pulldown via Hybridization followed by Mass Spectrometry (MORPH-MS) [37] employ antisense oligonucleotide probes to enrich endogenous RNAs together with associated proteins from crosslinked cells, as illustrated in Figure 2A. These methods are well suited for systematic interactome discovery and have been extensively applied to lncRNAs, viral RNAs, and chromatin-associated transcripts [31,32,37,38], although fixation-based workflows may capture indirect or proximity-driven interactions.

Proximity labeling-based RNA-centric approaches extend interactome mapping in vivo (Figure 2B) by enzymatically tagging proteins located within nanometer-scale distance of a target RNA. Platforms such as RaPID [25], BioID/TurboID [39,40], APEX/APEX2 [41,42], RNA-BioID [43], and Hybridization Proximity followed by Mass Spectrometry (HyPro-MS) [44,45] operated in proximity-labeling mode have proven effective for capturing dynamic, transient, and compartment-specific RPIs that are frequently lost in classical pulldown assays. Because these methods label spatial neighborhoods rather than direct binding interfaces, integration with orthogonal validation approaches is often required.

More recently, CRISPR-assisted RNA targeting systems (Figure 2C) based on catalytically inactive Cas13 variants have enabled programmable interrogation of endogenous transcripts without the need for extensive RNA tagging or overexpression. Techniques such as CRISPR-assisted RNA–protein interaction detection (CARPID) [46], CRISPR-Based RNA Proximity Proteomics (CBRPP) [47], and related dCas13-guided proximity-labeling platforms including CRISPR-based RNA-United Interacting System (CRUIS) [48], dCas13–APEX2 [49] and RNA proximity labeling combined with crosslinking immunoprecipitation (RPL-CLIP) [50] allow transcript-specific recruitment of labeling enzymes directly to native RNAs in vivo, offering increased flexibility, scalability, and multiplexing potential.

Overall, RNA-centric approaches provide complementary strategies for mapping RPI landscapes, and their integration with proteomics, imaging, and sequencing technologies has significantly improved the resolution and biological relevance of RPI studies [20,30,51,52].

#### 2.1.1. Hybridization-Based Pulldown Platforms

Hybridization-based RNA capture approaches marked an important turning point in the study of RPIs by enabling direct analysis of RNA-associated complexes within their native cellular context. Early implementations of these strategies used cell-permeable antisense DNA or nucleic acid analog probes conjugated to photoactivatable crosslinkers, allowing proteins in direct physical contact with a target RNA to be covalently trapped upon ultraviolet (UV) irradiation. UV exposure at 254 nm, which corresponds to peak nucleic acid absorption, promotes zero-distance crosslinking events, resulting in covalent bonds that form only at sites of direct RNA–protein contact. This high spatial specificity permits stringent purification under denaturing conditions and substantially reduces nonspecific protein carryover [30,53,54,55]. Despite these advantages, UV-based crosslinking is intrinsically inefficient, typically capturing only a small fraction of RNA-bound proteins. In practice, this limitation is compounded by shallow tissue penetration, sensitivity to irradiation conditions, and the narrow range of reactive amino acid–nucleobase chemistries, all of which restrict yield and scalability [30,53,54,55].

##### UV Crosslinking-Based Hybridization Approaches

As a result, UV-dependent methods tend to prioritize interaction specificity over proteome-wide coverage. Building on this foundation, the iDRiP strategy integrates UV crosslinking with pools of short, biotinylated antisense oligonucleotides tiled across the RNA of interest [35,36]. This design enables selective enrichment of proteins that directly contact the target RNA, including transient or low-affinity interactors that may dissociate during non-covalent purification workflows. While iDRiP provides high molecular resolution, its implementation requires extensive optimization of probe tiling, hybridization conditions, and crosslinking efficiency, increasing both experimental complexity and assay duration [35,36,56].

##### Chemical Crosslinking-Based Hybridization Approaches

An alternative and more widely adopted class of hybridization capture methods relies on chemical crosslinking agents, most commonly formaldehyde or glutaraldehyde, which generate reversible covalent bridges between nucleophilic groups in proteins and nucleic acids. This chemistry underlies platforms such as ChIRP [31] and CHART [32,33], which stabilize RNA–protein assemblies prior to purification. These approaches are particularly effective for preserving higher-order RNP complexes and RNA–chromatin interactions within intact cells [37]. Within this framework, tiled antisense oligonucleotide capture has emerged as a powerful strategy for RNA-centric interactome analysis. Pools of short, biotinylated DNA probes distributed along the length of a target transcript promote uniform hybridization, improving enrichment efficiency while minimizing positional bias. When applied to chemically crosslinked lysates, this strategy enables recovery of endogenous RNP assemblies that include both direct RBPs and spatially proximal chromatin-associated factors, facilitating comprehensive interrogation of RNA-associated protein networks [31,37].

Chromatin Isolation by RNA Purification followed by Mass Spectrometry (ChIRP-MS) further extends these approaches to transcript-centered, proteome-scale identification of RNA-associated proteins [57]. A major strength of this workflow lies in its reliance on conventional formaldehyde crosslinking and flexible probe design, which together support broad applicability across cell types, RNA classes, and experimental systems. Using this strategy, ChIRP-based studies have characterized protein interactomes associated with chromatin-regulatory lncRNAs involved in epigenetic control and nuclear organization, viral RNAs that recruit host RBPs to support replication or immune evasion, and nuclear-retained transcripts that function as structural scaffolds within the nucleus [31,58,59,60,61,62,63,64,65,66].

The versatility of these methods is closely linked to the properties of formaldehyde crosslinking itself. Unlike UV-induced crosslinking, which selectively stabilizes direct RNA–protein contacts, formaldehyde preserves both direct interactions and spatially proximal associations within intact RNP assemblies. This expanded interaction footprint enables more comprehensive profiling of large RNA–protein complexes, particularly those associated with chromatin or nuclear architecture [67,68,69]. However, this same feature reduces spatial resolution and increases the likelihood of recovering indirectly associated or nonspecific proteins. Consequently, interpretation of ChIRP-derived interactomes requires stringent experimental controls and orthogonal validation to distinguish functionally meaningful RBPs from proximity-driven bystanders [67,68].

Elution strategies also influence capture specificity and recovery. CHART incorporates RNase H–mediated elution following prior mapping of RNA accessibility, which improves probe placement accuracy and reduces off-target hybridization but adds additional experimental steps and limits throughput [32,33]. In contrast, RAP-MS typically employs strand displacement or denaturing elution strategies that favor high recovery, albeit at the cost of increased optimization to balance sensitivity and background enrichment [34].

##### Advanced Hybridization Approaches

To reduce probe synthesis costs and streamline workflows, MORPH-MS introduces a modular probe architecture in which a single biotinylated “collector” oligonucleotide recruits a pool of unmodified antisense probes tiled along the target RNA. This design substantially lowers reagent costs and labor requirements while maintaining enrichment efficiencies comparable to conventional tiled probe approaches. MORPH-MS is compatible with formaldehyde crosslinking to capture both direct and proximal interactors and can be adapted for UV crosslinking to enhance specificity toward direct RBPs. Validation studies have demonstrated robust and reproducible recovery of RNA-associated protein networks from diverse targets, including chromatin-associated lncRNAs such as NEAT1, underscoring its scalability and versatility [37].

Despite these improvements, MORPH-MS and related fixed-cell hybridization-based pulldown methods are generally best suited for moderately long transcripts, as probe length constraints limit their applicability to very short RNAs. In addition, the interactomes recovered by these approaches often include a mixture of functional and incidental associations, reinforcing the need for complementary biochemical, genetic, or imaging-based validation. Integration with quantitative proteomic strategies, such as SILAC or isobaric labeling, further enhances interpretability by enabling comparative analysis across biological conditions [37].

Methodological differences among hybridization-based capture platforms extend to probe length, hybridization stringency, and elution chemistry. Short tiled DNA probes (typically 20–25 nucleotides), commonly used in ChIRP and iDRiP workflows, offer cost-effective and flexible targeting but may exhibit reduced duplex stability under stringent wash conditions, particularly when applied to low-abundance or highly structured RNAs. In contrast, longer antisense probes (approximately 90–120 nucleotides), as employed in RAP-MS, form more stable RNA–DNA hybrids that tolerate harsher purification conditions, resulting in improved signal-to-noise ratios and higher-confidence identification of RNA-associated proteins [31,34,35,36,67]. These gains are offset by increased synthesis costs, greater technical demands, and higher starting material requirements [34,35,36].

Collectively, the choice of RNA hybridization capture strategy requires careful balancing of spatial resolution, interaction specificity, proteome coverage, throughput, material requirements, and cost (Table 1). UV-dependent approaches such as iDRiP offer exceptional precision for identifying direct and transient RBPs but are constrained by low yield and technical sensitivity. Formaldehyde-based methods, including ChIRP and related platforms, enable broader interactome discovery at the expense of spatial specificity. Recent innovations such as MORPH-MS partially mitigate longstanding technical and financial barriers associated with tiled probe hybridization, expanding access to scalable RNA-centric interactome mapping across diverse biological contexts [30,37,53,54,55,56,68].

#### 2.1.2. Protein Tagging by Proximity Labeling

Proximity-dependent protein labeling approaches have emerged as a transformative class of RNA-centric methodologies by enabling systematic identification of RNA-associated proteins directly within living or minimally perturbed cellular environments. Classical strategies for mapping RPIs, including antisense hybridization pulldowns and UV- or chemically induced crosslinking approaches, have been instrumental in defining the composition of RNP complexes. However, these techniques often suffer from inherent limitations, such as low crosslinking efficiency, loss of transient or weak interactions, limited spatial resolution, and perturbations introduced by harsh experimental conditions. Proximity labeling methods address many of these constraints by shifting the analytical paradigm from covalent trapping of direct RNA–protein contacts to enzymatic tagging of proteins that reside within a defined nanoscale environment surrounding a target RNA. This strategy enables capture of interaction landscapes in a manner that better reflects physiological organization and dynamic regulation of RNA-associated protein networks [70,71].

A defining feature of proximity labeling approaches is their reliance on engineered enzymes capable of covalently modifying nearby proteins in situ. These enzymes are positioned in close spatial proximity to the RNA of interest or to RNA-associated molecular scaffolds, allowing selective labeling of proteins within a typical radius of approximately 10–20 nm. Because labeling occurs in living cells or under minimally disruptive conditions, proximity labeling is uniquely suited to capture dynamic, transient, or low-affinity interactions that are frequently lost during conventional biochemical purification. This capability has proven particularly valuable for studying RNA-mediated regulatory processes such as co-transcriptional RNA processing, subcellular RNA trafficking, stress-induced RNP remodeling, and viral RNA–host protein interactions, where interaction kinetics and spatial context are critical determinants of function [70,71,72].

##### Hybridization-Coupled Proximity Labeling Approaches

One of the most notable recent advances in RNA-centric proximity labeling is the development of hybrid strategies that combine the sequence specificity of antisense RNA targeting with enzymatic protein tagging, while avoiding the need for genetic manipulation of the experimental system. HyPro-MS exemplifies this class of approaches (Figure 2B) by integrating antisense oligonucleotide hybridization with proximity-dependent labeling chemistry [44,45]. In this strategy, digoxigenin-labeled antisense DNA probes are designed to hybridize specifically to a target RNA within formaldehyde-fixed and permeabilized cells. Following hybridization, a recombinant fusion protein consisting of the APEX2 peroxidase and a high-affinity digoxigenin-binding domain is recruited to the RNA–probe complex. Upon activation with biotin-phenol and hydrogen peroxide, APEX2 generates short-lived biotin-phenoxyl radicals that rapidly label proteins in the immediate vicinity of the targeted RNA [44,73,74].

This hybridization–proximity design enables transcript-specific labeling of RNA-associated proteins without requiring ectopic expression of RNA tags or fusion proteins, making HyPro-MS particularly attractive for applications in primary cells, difficult-to-transfect systems, and clinical samples where genetic manipulation is impractical or undesirable. HyPro-MS has been successfully applied to profile interactomes of both abundant and low-copy RNA species, including 45S pre-rRNA, nuclear-retained lncRNAs such as NEAT1 and PNCTR, and oncogenic mRNAs such as c-Myc [44,45]. These studies revealed spatially organized RPI networks that contribute to nuclear architecture, compartmentalization, and regulatory function, underscoring the utility of HyPro-MS for dissecting RNA biology within complex subcellular environments [44,73,75].

Despite its broad applicability, HyPro-MS also introduces technical considerations that require careful optimization. Effective antisense probe design is critical, particularly for RNAs with extensive secondary structure, repetitive sequences, or limited accessibility. In addition, while formaldehyde crosslinking preserves many physiologically relevant interactions, it can also stabilize indirect or proximity-driven associations, complicating the interpretation of recovered interactomes. Suboptimal crosslinking may either restrict interactome recovery or increase background noise. Consequently, rigorous experimental controls, replicate analyses, and orthogonal validation approaches remain essential for distinguishing functional RBP from spatially adjacent bystanders [44,74].

##### Genetically Encoded RNA Tagging Approaches

A distinct RNA-centric proximity labeling strategy seeks to preserve native RNA expression, localization, and regulatory control by directly modifying endogenous RNA loci. RNA-BioID represents a prominent example of this approach [43]. Rather than relying on overexpression of tagged RNAs, RNA-BioID employs genome-editing technologies, most commonly CRISPR/Cas9, to insert RNA aptamer sequences into the genomic locus encoding the target RNA. The engineered RNA is subsequently recognized by a corresponding aptamer-binding protein fused to a biotin ligase (Figure 2B). Upon biotin supplementation, proteins located within approximately 10 nm of the RNA are biotinylated and recovered for mass spectrometry-based identification. This approach has enabled high-throughput profiling of RNA-associated proteomes in live cells and facilitated studies of co-transcriptional processing, splicing, and RNA localization with minimal disruption to native cellular processes [43,70,71,72,73].

By enabling proximity labeling at endogenous RNA expression levels, RNA-BioID minimizes artifacts associated with RNA overexpression and preserves physiological regulation of transcription, processing, and localization. This strategy has demonstrated high sensitivity in mapping RNA-associated protein networks, exemplified by studies of the β-actin mRNA interactome [43]. In this context, RNA-BioID uncovered dynamic and stimulus-dependent recruitment of regulatory proteins during cell migration, revealing numerous previously unrecognized RBPs and regulatory factors involved in spatial and temporal RNA control. These findings highlight the capacity of RNA-BioID to capture both stable core interactors and condition-specific associations that would be difficult to detect using fixed-cell pulldown approaches [43,70,73].

Nevertheless, RNA-BioID also introduces specific challenges that must be considered during experimental design and interpretation [43]. Insertion of aptamer sequences may alter RNA folding, stability, or interaction potential, potentially affecting RNA function. Furthermore, the requirement for genome editing and expression of fusion proteins limits applicability in certain experimental systems, including primary cells, non-model organisms, or clinical samples. Optimization of aptamer copy number, fusion protein architecture, and expression levels is therefore essential to minimize background labeling, avoid cytotoxicity, and prevent mislocalization artifacts [76]. As with other proximity-based approaches, RNA-BioID labels proteins based on spatial proximity rather than direct physical contact, necessitating complementary validation strategies to confirm *bona fide* RPIs [43,49,70,73].

##### Enzymatic Platforms for Proximity Labeling

At a mechanistic level, most RNA-centric proximity labeling strategies are built upon two enzymatic platforms: promiscuous biotin ligases and engineered peroxidases. Promiscuous biotin ligases, most notably the mutant *Escherichia coli* BirA enzyme, form the foundation of BioID-based proximity labeling (Figure 2B) [39,42]. BirA-based approaches enable labeling of nearby proteins over extended time scales, facilitating detection of weak or transient interactions [39,70]. However, prolonged labeling can increase background signal, and careful optimization of expression levels and controls is required. As a result, orthogonal validation methods such as RIP or CLIP-based assays are often used to confirm RPIs [30,71,77,78].

Engineered peroxidases such as APEX and APEX2 provide an alternative enzymatic platform with markedly faster labeling kinetics [40,41,42,79]. Upon activation with biotin-phenol and hydrogen peroxide, APEX enzymes generate highly reactive biotin-phenoxyl radicals that label nearby proteins within seconds to minutes [42]. This rapid labeling allows interrogation of RPI landscapes with high temporal resolution and makes APEX particularly valuable for studying rapidly changing RNA-associated assemblies, such as stress granules, transcriptional hubs, or subcellular RNA localization events [40,42]. APEX-based RNA-centric strategies have been successfully applied to map RNA-associated proteomes at defined subcellular locations, including organellar membranes and nuclear compartments.

However, the chemistry underlying APEX labeling introduces additional considerations. The generation of reactive oxygen species during labeling can induce cellular stress or toxicity, particularly in sensitive cell types or with prolonged exposure [42,79]. Inaccurate targeting of the enzyme may also result in nonspecific labeling of unrelated proteins. Consequently, rigorous construct validation, optimization of labeling duration and reagent concentrations, and inclusion of stringent controls are essential to minimize background and ensure reproducibility [70].

##### RNA-Adapted Proximity Labeling Systems

Building upon these enzymatic foundations, RNA-adapted proximity labeling platforms have been developed to directly interrogate RNA-centered interactomes [70,72]. The RaPID system represents a widely used example of this class [25]. RaPID exploits the high-affinity interaction between the λN peptide and BoxB RNA stem-loop structures to recruit a biotin ligase to a specific RNA transcript. In this approach, the RNA of interest is engineered to contain BoxB motifs, while the λN peptide is fused to a biotin ligase such as BirA or the faster-acting BASU variant. Upon co-expression and biotin supplementation, proteins in close proximity to the RNA are selectively biotinylated in living cells (Figure 2B) [25].

RaPID enables real-time profiling of RNA-associated protein networks under physiological conditions and has been applied to diverse biological contexts, including viral infection, cellular stress responses, and disease-associated RNA structural variants. By enriching and identifying biotinylated proteins via mass spectrometry, RaPID reveals comprehensive RNA-centered interactomes, including transient or low-affinity interactions that may be missed by conventional approaches [25,73]. A key advance in the RaPID platform was the development of the BASU biotin ligase, derived from Bacillus subtilis, which exhibits enhanced catalytic activity relative to BirA. BASU enables efficient labeling within minutes and improves signal-to-noise ratios, supporting high-temporal-resolution interactome mapping while reducing background labeling [25].

Despite its utility, RaPID has notable limitations. Ectopic expression of BoxB-tagged RNAs and λN-fused ligases may perturb endogenous RNA abundance, localization, or structure, potentially generating non-physiological interactions. Moreover, RaPID does not provide nucleotide-level binding resolution and cannot distinguish direct RBPs from indirectly associated factors within the labeling radius. Analysis of low-abundance endogenous RNAs often requires genome editing to introduce BoxB motifs at native loci, adding technical complexity. Appropriate negative controls, such as scrambled RNA motifs or ligase-only constructs, are therefore essential for accurate interpretation [25,30,71].

Collectively, proximity labeling-based RNA-centric approaches have transformed the study of RPIs by enabling analysis in living or minimally perturbed systems and capturing interaction dynamics beyond direct physical contacts (Table 2). Hybrid non-genetic platforms such as HyPro-MS expand applicability to challenging systems, endogenous RNA tagging strategies like RNA-BioID preserve physiological regulation, enzymatic platforms such as BioID and APEX offer complementary temporal resolution, and RNA-adapted systems like RaPID provide transcript-specific interactome mapping [25,39,42,43,44]. Together, these approaches complement hybridization-based pulldown and crosslinking strategies, forming a versatile toolkit for dissecting RNA-mediated regulation. As advances in enzyme engineering, RNA-targeting specificity, genome editing, and integrative validation strategies continue, proximity labeling is expected to play an increasingly central role in resolving the spatial and temporal complexity of RNA regulatory networks in both physiological and disease contexts [30,72,73].

#### 2.1.3. CRISPR-Assisted Proximity Labeling

CRISPR/Cas13–guided proximity labeling has emerged as a powerful strategy (Figure 2C) for mapping RPI landscapes directly within living cells, particularly for endogenous RNAs that are difficult to interrogate using hybridization-based pulldown or antibody-dependent approaches. By leveraging sequence-specific RNA recognition rather than RNA capture, these methods enable enzymatic labeling of RNA-proximal proteins under near-physiological conditions, thereby preserving native RNA expression levels, subcellular localization, and regulatory dynamics [49,80,81]. This capability establishes Cas13-based platforms as a core component of contemporary RNA interactomics, particularly for the interrogation of low-abundance, structurally complex, and highly dynamic transcripts.

At the core of these approaches is catalytically inactive Cas13 (dCas13), which retains RNA-binding and CRISPR RNAs (crRNA) processing activity while lacking nuclease function [49,81]. Fusion of dCas13 to proximity labeling enzymes or orthogonal tagging modules enables programmable recruitment of enzymatic activity to defined RNA regions, allowing spatially restricted labeling of RNA-associated protein neighborhoods. Unlike RNA tagging systems that uniformly decorate entire transcripts, Cas13-guided targeting permits region-specific interrogation, making it possible to dissect modular RNAs whose functional domains recruit distinct protein cohorts [49]. This feature has proven particularly informative for lncRNAs and circular RNAs that act as multifunctional scaffolds.

##### CARPID

Among the earliest and most widely adopted implementations is CARPID, which typically employs dCas13 fused to fast-acting biotin ligases such as BASU [42,80]. CARPID enables proximity-dependent labeling of proteins surrounding endogenous transcripts in living cells without the need for RNA modification or crosslinking, facilitating detection of weak, transient, or condition-specific interactions. Using this approach, researchers have mapped the interactomes of nuclear lncRNAs including XIST, MALAT1, DANCR, and TUG1, uncovering enrichment of chromatin remodelers, RNA helicases, splicing regulators, and genome stability factors such as hnRNPU, SPEN, DHX9, RPA32, and components of Polycomb and R-loop resolution machinery [42,73,80,81,82,83].

Beyond descriptive interactome mapping, CARPID has enabled functional insight into RNA-mediated regulatory pathways. For example, CARPID analysis of TUG1 revealed proximity to DHX9 and RPA32, linking this lncRNA to DNA replication stress and genome maintenance pathways [82,84,85]. Similarly, mapping of LETS1 and LITATS1 in breast cancer models identified signaling proteins such as TβRI, SMURF2, and NFAT5, providing mechanistic connections between RNA regulation and TGF-β-driven epithelial–mesenchymal transition [85,86]. Recent CARPID studies have further implicated lncRNA-associated interactomes in immune signaling pathways by identifying proximity of inflammatory RBPs and ubiquitin ligases during cytokine stimulation [85].

##### Variants and Extensions of Cas13-Guided Proximity Labeling

Several alternative Cas13-guided proximity labeling platforms extend this conceptual framework. The CRUIS couples dCas13 targeting with PUP-IT chemistry, enabling uridine tagging of RNA-proximal proteins and providing an orthogonal labeling strategy that reduces reliance on biotinylation alone [48]. This approach has been applied to nuclear RNAs residing in dense compartments, where biotin-based methods can be confounded by high background. Cas13–APEX2 implementations further enable rapid, time-compressed labeling windows, supporting capture of RNA-associated protein neighborhoods during fast cellular transitions [49]. For instance, Cas13–APEX2 has been used to monitor remodeling of stress-responsive mRNPs, revealing rapid recruitment of translational repressors, RNA helicases, and chaperones during stress granule assembly and disassembly [49].

CBRPP generalizes Cas13-guided proximity labeling by coupling dCas13 targeting to proximity biotinylation without RNA modification, allowing profiling of endogenous RNA interactomes across diverse RNA classes [47]. This strategy has been applied to nuclear-retained RNAs, low-copy lncRNAs, and structured RNAs that are poorly accessible to antisense probe-based pulldowns. More recently, CBRPP has been extended to enhancer RNAs (eRNAs), uncovering transient associations with transcriptional coactivators, mediator components, and chromatin remodelers at active regulatory loci.

Cas13-guided proximity labeling has also proven valuable in infectious disease contexts. CARPID-based mapping of the circular RNA ciTRAN revealed selective proximity to the splicing factor SRSF1, supporting a model in which ciTRAN acts as a molecular decoy to modulate HIV-1 transcription [73,80,82,87]. Additional Cas13-based studies targeting viral genomic and subgenomic RNAs have identified host factors involved in viral RNA stability, replication, and immune evasion, highlighting the adaptability of these platforms for pathogen–host interaction studies [88].

To partially bridge the gap between proximity-based neighborhood capture and direct interaction mapping, RPL-CLIP integrates Cas13-guided proximity recruitment with UV-induced crosslinking to stabilize direct RNA–protein contacts prior to purification [50]. By coupling guide-positioned labeling with nucleotide-resolution CLIP readouts, RPL-CLIP improves specificity for *bona fide* RNA binders while retaining the programmability of CRISPR-based targeting, making it particularly useful for validating candidate interactions identified by CARPID or CBRPP [40,46,47,80].

Despite their versatility, Cas13-assisted proximity labeling approaches require careful experimental design and interpretation. Guide RNA accessibility can be limited by RNA secondary structure or RBP occupancy, necessitating guide tiling, empirical validation, and expression tuning to achieve robust targeting [80,81]. Moreover, because proximity labeling reports spatial neighborhood rather than direct physical binding, proteins identified may reflect compartmental co-residence rather than *bona fide* RNA binders. Consequently, orthogonal validation using CLIP-based crosslinking, RNA pulldown, mutational analysis, or functional perturbation remains essential for mechanistic inference [68,73,80]. Comparative analyses further indicate that different proximity chemistries, such as biotin ligases, peroxidases, and PUP-IT, recover overlapping but non-identical RNA-associated proteomes, emphasizing that enzyme choice, labeling duration, and subcellular context strongly influence apparent interactomes [48].

When considered alongside other RPI methodologies, CRISPR-assisted proximity labeling occupies a complementary niche (Table 3). UV crosslinking approaches such as CLIP and iDRiP provide nucleotide-level resolution for direct RNA–protein contacts but are biased toward stable interactions and lack spatial context [68,73]. Hybridization-based pulldown strategies such as ChIRP and CHART (Figure 2A) excel at capturing large RNA-centered assemblies but are susceptible to indirect associations stabilized during fixation [31,32,68,73]. Cas13-guided proximity labeling trades atomic resolution for programmability, contextual fidelity, and in situ neighborhood capture, making it particularly well suited for dissecting dynamic RNA regulatory networks in living cells. Ongoing development of compact Cas13 variants, improved guide-design algorithms, and advanced delivery modalities is expected to further expand these approaches into primary cells, organoids, and in vivo models where conventional RPI methods remain challenging [89].

### 2.2. Protein-Centric Methods

Protein-centric strategies for RPI analysis adopt an inverse experimental logic compared with RNA-centric methods, using RBPs as the point of entry to define their associated RNA landscapes. These approaches directly link RNA binding to RBP function and regulatory outcomes. This perspective is particularly powerful because RBPs function as dynamic regulators of post-transcriptional gene expression, coordinating RNA splicing, localization, stability, translation, and decay across diverse cellular contexts [73,90].

A key advantage of protein-centric analysis is its ability to capture context-dependent RBP behavior. Many RBPs exhibit cell-type-specific, developmental, or stimulus-responsive binding profiles, and often act as hubs integrating signaling pathways with RNA metabolism [90,91,92]. Accordingly, approaches that resolve RBP–RNA interactions under native conditions are essential for understanding their full regulatory scope.

Protein-centric methods can be broadly classified into three complementary categories based on experimental design and resolution. High-throughput in vitro approaches, such as High-throughput RNA SELEX (HTR-SELEX; systematic evolution of ligands by exponential enrichment) and sequencing of RNA substrate specificity landscapes (SEQRS), are designed to define the intrinsic sequence and structural preferences of RBPs using synthetic RNA libraries, providing mechanistic insight into RNA recognition principles independent of cellular context [68,90]. While powerful for motif discovery, these approaches do not capture cellular complexity.

In contrast, immunoprecipitation-based methods, including RIP, CLIP and its derivatives (e.g., iCLIP, eCLIP), and linear amplification of complementary DNA ends and sequencing (LACE-seq), capture RBP–RNA complexes directly from cells, enabling transcriptome-wide identification of binding sites with high or nucleotide-level resolution [68,92,93]. Advances in crosslinking and library preparation have improved sensitivity and reduced background, enabling analysis of low-abundance RBPs and heterogeneous samples [93].

A third and increasingly important class comprises proximity-tagging approaches such as Targets of RNA-binding proteins Identified by Editing (TRIBE) and related enzymatic RNA-labeling strategies, which couple RBPs to catalytic domains that mark nearby RNAs in living cells [68,94]. Unlike crosslinking-based methods, proximity tagging does not require stable RBP–RNA complexes, making it well suited for capturing transient, low-affinity, or spatially restricted interactions. This has proven especially valuable in polarized cells and neuronal systems, where RBPs regulate local RNA translation in subcellular compartments that are difficult to isolate biochemically [68,93].

Overall, protein-centric approaches provide a complementary framework to RNA-centric strategies by defining how RBPs shape RNA fate and regulatory networks. While RNA-centric methods identify proteins associated with a transcript, protein-centric workflows establish how specific RBPs influence downstream regulatory programs [90,93]. Integrative analyses combining protein-centric data with transcriptomic and proteomic readouts further enable reconstruction of RBP-centered regulatory networks [91].

Protein-centric methods have also played a pivotal role in elucidating disease-associated RBP dysfunction. For example, CLIP-based mapping of FUS and TDP-43 has uncovered widespread alterations in splicing, RNA transport, and RNA stability pathways linked to amyotrophic lateral sclerosis and frontotemporal dementia [68,95,96]. Similarly, studies of HuR (ELAVL1) and IGF2BP family proteins have demonstrated how aberrant stabilization of oncogenic mRNAs contributes to tumor progression and therapy resistance [68,91]. Proximity-tagging approaches such as TRIBE have further revealed activity-dependent remodeling of RBP–RNA interactions in neuronal dendrites, providing mechanistic insight into synaptic plasticity and neurodevelopmental disorders [68,93].

With continued improvements in sensitivity, temporal resolution, and multi-omics integration, protein-centric methods are increasingly capable of capturing dynamic and low-abundance RPIs. These advances are enabling a shift from cataloging RBP targets toward understanding how RPI networks are rewired in response to physiological and disease-associated perturbations [90,91].

#### 2.2.1. In Vitro High-Throughput Analysis

One of the foundational strategies for dissecting RPI under controlled biochemical conditions is systematic evolution of ligands by exponential enrichment (SELEX), commonly referred to as RNA-SELEX when applied to RNA molecules, an in vitro selection framework designed to identify RNA sequences preferentially bound by a given protein [19,97,98]. Rather than interrogating cellular binding events, RNA-SELEX isolates the intrinsic affinity of RBPs by subjecting large pools of randomized RNA molecules to iterative rounds of binding, partitioning, and amplification. Through successive enrichment cycles, this approach yields RNA ligands with increasing affinity while simultaneously revealing the sequence and structural features that underlie molecular recognition. The iterative selection process enriches high-affinity binders while progressively eliminating non-specific or weak interactions, thereby enhancing binding specificity. In addition, analysis of the selected RNA pools provides insight into minimal binding motifs and sequence constraints required for stable RBP–RNA complex formation.

A major conceptual contribution of early SELEX studies was the realization that RNA recognition often depends on combinatorial features, including both primary sequence motifs and higher-order secondary structures, rather than simple linear consensus elements [97,98]. Although classical SELEX experiments were experimentally demanding and limited in scale, they established a mechanistic framework that continues to inform models of RBP–RNA recognition [19,97,98]. Importantly, these early datasets also provided some of the first evidence that RBPs can tolerate substantial sequence degeneracy while maintaining binding specificity, a property that helps explain their ability to regulate diverse RNA substrates in vivo.

**Figure 3:**
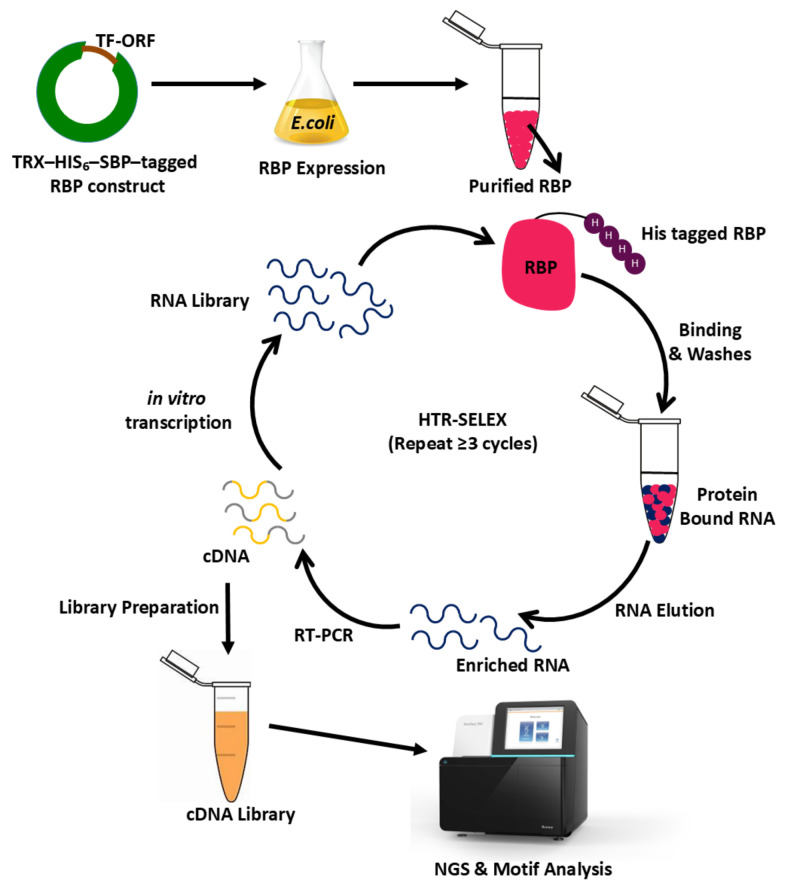
Schematic illustration of in vitro protein-centric approaches. High-throughput RNA systematic evolution of ligands by exponential enrichment (HTR-SELEX). Purified RNA-binding proteins are incubated in vitro with a randomized RNA library, followed by washing to remove unbound RNAs, recovery of protein-associated RNAs, cDNA amplification, and high-throughput sequencing to quantitatively map RNA substrate selectivity landscapes.

The introduction of NGS transformed SELEX from a low-throughput biochemical assay into a scalable discovery platform capable of surveying millions of RNA variants in parallel [99,100,101,102]. Both HTR-SELEX, an advanced sequencing-coupled variant of RNA-SELEX, as illustrated in Figure 3, which schematically represents selection-based enrichment of RNA ligands followed by cDNA generation and sequencing-based analysis of RBP binding preferences, and SEQRS integrate classical enrichment workflows with deep sequencing to generate quantitative binding landscapes for RBPs, providing mechanistic insight into RNA recognition principles independent of cellular context [19,99,100,103,104]. While both approaches follow similar selection logic, they differ in library design: SEQRS typically employs RNA pools with ~20 randomized nucleotides, whereas HTR-SELEX expands this to ~40 nucleotides, substantially increasing the combinatorial space of possible sequence and structural motifs [99,100]. This expanded diversity has proven critical for capturing RBPs that recognize long or structured RNA elements that may be underrepresented in shorter libraries [100,104].

Large-scale application of these high-throughput SELEX variants has revealed unexpected complexity in RBP binding behavior. For example, HTR-SELEX analyses across dozens of human RBPs demonstrated that many proteins bind multiple distinct RNA motifs, including combinations of linear sequences and structured elements, rather than a single invariant consensus [100]. This versatility is consistent with the multifunctional roles of RBPs in splicing, RNA localization, and turnover, where context-dependent binding is often required [19]. More recent analyses have further shown that some RBPs display condition-dependent motif preferences in vitro, suggesting that subtle changes in protein conformation or cofactor availability can reshape binding landscapes [105].

Beyond mapping individual RBPs, SELEX-derived datasets have increasingly been integrated with computational modeling, structural biology, and comparative genomics [105,106]. These integrative efforts have enabled the construction of predictive models linking RNA sequence and structure to binding affinity, providing a bridge between biochemical specificity and cellular regulation [105]. Notably, machine-learning approaches trained on HTR-SELEX data have begun to predict RBP binding sites in vivo with increasing accuracy, highlighting the growing synergy between high-throughput in vitro assays and computational biology [107].

High-throughput SELEX approaches have also found application in translational and virological research [108]. Amid the COVID-19 pandemic, HTR-SELEX emerged as a powerful approach for defining the RNA-binding landscapes of coronavirus nucleocapsid proteins, yielding critical insights into viral genome packaging and replication strategies [19,109]. Such studies underscore the utility of SELEX-based platforms for rapidly characterizing RPIs in emerging pathogens. More broadly, SELEX-derived binding models are now being explored as a foundation for therapeutic design, including the development of RNA-based decoys and small molecules that disrupt pathological RBP–RNA interactions [110].

Despite their power, it is important to recognize that SELEX-based methods capture intrinsic binding preferences in isolation and do not account for cellular variables such as RNA competition, subcellular localization, or post-translational modification of RBPs [19]. As such, high-throughput in vitro analyses are most informative when interpreted alongside in vivo protein-centric or RNA-centric interaction data. Together, these complementary approaches provide a multiscale view of RNA–protein recognition, spanning biochemical specificity and cellular function.

#### 2.2.2. In Vivo Approaches

**Figure 4:**
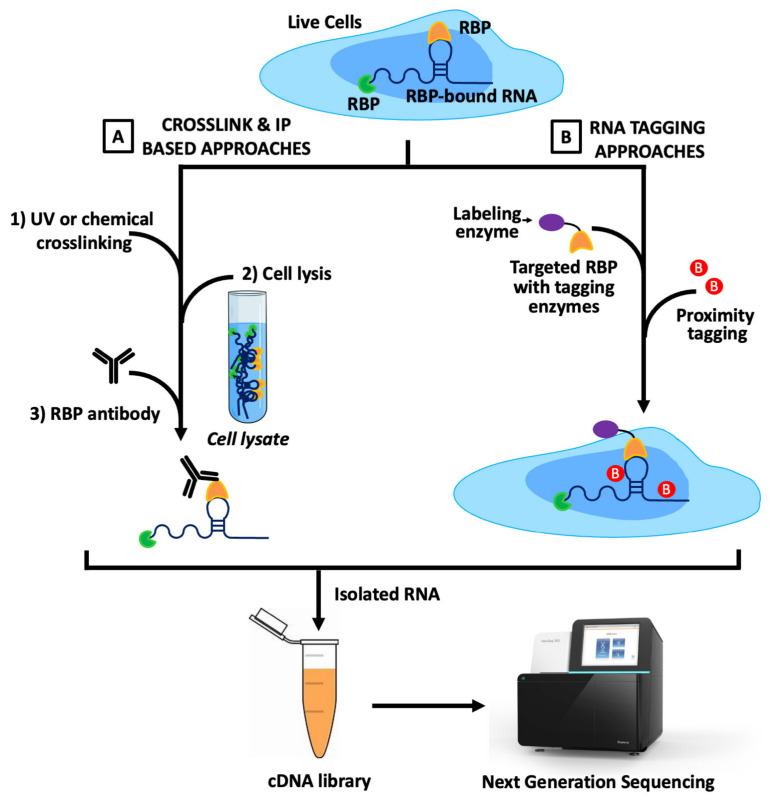
Schematic illustration of in vivo protein-centric approaches. (**A**) In crosslinking and immunoprecipitation-based methods, RNA–protein complexes formed in live cells are first stabilized by UV or chemical crosslinking, followed by cell lysis. Antibodies are then added to bind the RBP, enabling immunoprecipitation and isolation of associated RNAs for downstream analysis. (**B**) In proximity-based RNA tagging approaches, an RBP is fused to or recruits a labeling enzyme that marks RNAs in the immediate vicinity of the RBP within living cells, enabling recovery and sequencing-based identification of RBP-bound transcripts.

In vivo protein-centric strategies for studying RPIs mainly rely on crosslinking-based methods and proximity-tagging approaches (Figure 4A,B) [53]. Immunoprecipitation-based strategies often employ UV or chemical crosslinkers to stabilize native RNA–protein complexes, after which the RNA bound to the target protein is captured using specific antibodies and subsequently identified by sequencing (Figure 4A) [68]. By contrast, RNA tagging approaches fuse RBPs to RNA-modifying enzymes such as ADAR, APOBEC1, PUP, or APEX2, as illustrated in the RNA tagging panel of Figure 4B, which introduce distinct marks on nearby transcripts [53]. These labeled RNAs are then selectively enriched, prepared into sequencing libraries, and analyzed to reveal the interaction landscape, as depicted in Figure 4 [68]. Together, crosslinking and proximity-tagging strategies enable the capture of both stable and transient RBP–RNA interactions directly in living cells, offering complementary insights into post-transcriptional regulatory networks [53,111].

##### Immunoprecipitation-Based RNA Identification

In vivo protein-centric techniques such as RIP or co-immunoprecipitation, as illustrated in Figure 4A, have been foundational for mapping the landscape of RNA molecules associated with a given RBP across the transcriptome [53]. This approach does not require any prior information about the identity or sequence of potential RNA targets, allowing the discovery of both known and novel RBP–RNA interactions in a largely unbiased manner. In a typical protocol, cells are lysed under conditions that preserve endogenous RNP complexes, followed by immunoprecipitation of the RBP of interest using a highly specific antibody, as depicted in Figure 4A [53]. RNAs bound within the isolated complexes are subsequently purified and identified using downstream methods such as RT-qPCR, microarrays, or high-throughput sequencing, resulting in a comprehensive inventory of RBP-associated transcripts. Although traditional RIP methods excel at capturing a global view of RBP–RNA interactions, they typically do not pinpoint the precise binding site on the RNA; recent advances that incorporate crosslinking or digestion-optimized protocols enable more refined mapping, offering improved resolution for understanding protein–RNA binding at the nucleotide level [53,112].

RIP-Seq and CLIP-Based Approaches

RNA immunoprecipitation followed by sequencing (RIP-seq) represents one of the most widely used protein-centric strategies for identifying RNA populations associated with a specific RBP in vivo [68,113]. In this approach, endogenous RNP complexes are enriched by antibody-mediated immunoprecipitation of the target RBP from cell lysates, and co-purifying RNAs are subsequently identified by high-throughput sequencing [53]. Compared with more technically demanding RNP mapping methods, RIP-seq is considered experimentally accessible, as it does not require specialized crosslinking equipment, metabolic labeling, or complex library preparation workflows [68].

A defining feature of RIP-seq is its flexibility in preserving native RNA–protein assemblies. Cell lysis conditions can be tuned to maintain physiological RNP integrity, enabling recovery of both coding and non-coding RNA targets that interact with the RBP under steady-state conditions [53]. This relatively gentle purification can be advantageous for detecting moderately stable or condition-dependent associations that may be disrupted by harsher crosslinking-based approaches, particularly for RBPs involved in RNA turnover or transport. To improve interaction specificity, RIP-seq workflows are often supplemented with UV or chemical crosslinking, most commonly formaldehyde, which stabilizes RPIs prior to immunoprecipitation [68]. However, careful optimization of crosslinking conditions is essential, as excessive crosslinking can compromise RNA integrity or epitope recognition, whereas insufficient crosslinking increases susceptibility to nonspecific RNA carryover [68].

Despite its utility for global target discovery, RIP-seq has several inherent limitations. One major constraint is its dependence on antibody quality and specificity, which can vary widely among RBPs and limit scalability across large protein families [53]. In addition, RIP-seq typically reports transcript-level enrichment rather than precise binding locations, making it difficult to distinguish direct RNA recognition from indirect association mediated by multi-protein complexes, particularly for RBPs embedded within large RNP assemblies [51]. As a result, traditional RIP-seq data are often best interpreted as a survey of potential RNA targets rather than definitive binding maps.

To address these resolution limitations, several methodological refinements have been developed. By combining crosslinking with controlled RNase digestion, optimized RIP variants facilitate precise, nucleotide-resolution identification of RBP interaction sites [15,20,23,114]. Comparative analyses indicate that combining RIP-seq for transcript-level discovery with CLIP validation substantially improves confidence in identifying biologically relevant RPIs, particularly for RBPs with broad or heterogeneous RNA binding profiles.

RIP-seq has also proven versatile in profiling interactions with diverse RNA classes, including miRNAs, lncRNAs, and other regulatory transcripts, highlighting its utility beyond mRNA-centric studies [113]. When integrated with transcriptomic or functional perturbation data, RIP-seq can further help define RBP-centered regulatory networks and link RNA binding to downstream effects on gene expression, reinforcing its continued relevance within the expanding RPI methodological landscape. Nonetheless, investigators must remain mindful of indirect RNA pulldown and background enrichment, emphasizing the importance of appropriate controls and orthogonal validation strategies when interpreting RIP-seq datasets [68,113].

Among protein-centric strategies for defining RPIs, crosslinking-based methodologies collectively referred to as CLIP have become indispensable for mapping direct RNA–protein contacts within living cells [20,78,115]. Unlike native immunoprecipitation approaches, CLIP introduces an in vivo UV crosslinking step that covalently stabilizes RNA–protein complexes prior to cell lysis, thereby minimizing post-lysis reassociation artifacts and enabling interrogation of direct molecular contacts. This conceptual shift from enrichment of associated transcripts to stabilization of physical binding interfaces distinguishes CLIP from classical RIP-based methods.

The core principle underlying CLIP relies on UV irradiation, typically at 254 nm, which induces covalent bonds between RNA nucleotides and amino acid residues positioned at direct contact sites [20,78,115]. This crosslinking event acts as a molecular footprint, preserving native RPIs through subsequent purification and stringent washing steps. After immunoprecipitation of the RBP of interest, limited RNase digestion is used to trim bound RNA fragments, generating short RNA tags that correspond to binding sites [116]. High-throughput sequencing of these fragments, commonly referred to as HITS-CLIP or CLIP-seq, enables transcriptome-wide reconstruction of RBP binding landscapes [116,117].

One of the major strengths of CLIP lies in its capacity to resolve RNA-binding events with far greater precision than native RIP approaches. However, early implementations of HITS-CLIP were constrained by stochastic crosslinking efficiency and difficulty in pinpointing exact crosslink positions [117,118]. To overcome these limitations, successive methodological refinements introduced distinct improvements in resolution and sensitivity. Photoactivatable Ribonucleoside-Enhanced CLIP (PAR-CLIP) enhanced crosslinking efficiency by incorporating photoreactive nucleoside analogs such as 4-thiouridine (4SU) into nascent transcripts [118,119]. Upon UV irradiation at 365 nm, these analogs crosslink more efficiently and generate diagnostic T-to-C mutations at crosslink sites during reverse transcription, providing higher-confidence identification of binding positions [120]. This innovation significantly improved signal-to-noise ratios and enabled robust detection of dynamic regulatory networks, particularly in studies of RNA decay and post-transcriptional regulation [120,121]. iCLIP addressed a different technical limitation: reverse transcription frequently terminates at crosslinked nucleotides, producing truncated cDNAs [23]. Rather than treating these truncations as artifacts, iCLIP exploits them as informative signals to directly pinpoint protein–RNA contact sites at single-nucleotide resolution [23,122,123]. This refinement has been especially valuable in dissecting binding architectures of splicing regulators and in mapping motif positioning across complex transcript isoforms. Enhanced CLIP (eCLIP), developed and standardized under the ENCODE consortium, focused on improving scalability, reproducibility, and quantitative rigor [52,124]. By incorporating size-matched input controls and streamlined library preparation workflows, eCLIP reduced background artifacts and enabled systematic profiling of dozens of RBPs across multiple cell types [15,52,124]. The introduction of standardized quality-control metrics and cross-replicate validation frameworks under ENCODE has further elevated CLIP from a specialized technique to a large-scale functional genomics platform, enabling direct comparison of RBP binding landscapes across conditions and cell types [52].

Collectively, the CLIP family of approaches provides complementary advantages depending on experimental objectives. HITS-CLIP offers robust global mapping of RBP–RNA interactions; PAR-CLIP increases crosslink specificity and diagnostic confidence; iCLIP delivers nucleotide-level precision; and eCLIP enables high-throughput comparative analysis across large RBP cohorts [23,52,118,124]. More recently, integrative analyses combining CLIP-derived binding maps with RNA-seq, ribosome profiling, and chromatin accessibility datasets have begun to reveal how RBPs coordinate multi-layered regulatory programs rather than acting in isolation, underscoring the expanding systems-level impact of CLIP technologies [115].

Importantly, while CLIP-based methods excel at identifying direct contact sites, they remain influenced by crosslinking biases, sequence context, and cellular UV exposure constraints [118,120]. Crosslink efficiency varies among RBPs and RNA sequence contexts, leading to potential underrepresentation of certain interaction classes, and careful experimental design remains essential to ensure interpretability. As such, the choice among HITS-CLIP, PAR-CLIP, iCLIP, and eCLIP should be guided by the balance between desired resolution, crosslinking efficiency, sample availability, and experimental scale (Table 4).

Although CLIP-based methods have revolutionized transcriptome-wide mapping of RPIs, their practical implementation remains constrained by relatively high cell number requirements and amplification-associated bias [118]. These constraints become particularly limiting when studying rare cell populations, primary tissues, or developmental transitions where only limited material is available. To address these challenges, LACE-seq was developed as a low-input, high-resolution alternative that preserves the nucleotide precision of CLIP while substantially reducing input requirements [111,125].

LACE-Seq: A Low-Input CLIP-Derived Approach

Rather than modifying crosslinking chemistry, LACE-seq re-engineers the amplification strategy. As in CLIP, cells are UV crosslinked to stabilize direct RNA–protein contacts, ensuring that interaction footprints reflect in vivo binding events [125]. However, the distinguishing innovation lies in the exploitation of reverse transcriptase (RT) stalling at crosslink sites as a high-fidelity molecular marker of protein–RNA contact [125]. Following immunoprecipitation and controlled RNA fragmentation, adaptor ligation and primer design enable RT to initiate from defined RNA ends, and the resulting truncation events are preserved during library preparation. Unlike PCR-heavy CLIP workflows, LACE-seq employs linear amplification via in vitro transcription, thereby minimizing exponential amplification bias and improving quantitative representation of binding frequency.

This architecture enables mapping of protein–RNA contact sites with single-nucleotide precision from input material orders of magnitude lower than that required for classical CLIP [125]. Recent benchmarking studies have shown that LACE-seq can generate high-quality binding maps from fewer than one thousand cells, with reproducibility comparable to eCLIP datasets derived from millions of cells. This sensitivity makes the method particularly well suited for systems in which cellular heterogeneity or limited sample availability would otherwise preclude high-resolution RBP mapping.

While early applications emphasized germline and early embryonic systems, the utility of LACE-seq extends beyond developmental biology. In mammalian oocytes and early embryos, LACE-seq has elucidated binding landscapes of RBPs such as AGO2, MILI, DDX4, PTBP1, EIF4E1B, NAT10, LSM14B, and ZAR-family proteins, revealing coordinated regulation of maternal mRNA storage, translation, and clearance [125,126,127,128,129,130]. These studies highlighted how RNA modification enzymes such as NAT10 and translational regulators like EIF4E1B converge to orchestrate zygotic genome activation and early developmental competence [126,128].

More recently, LACE-seq has been applied to neural progenitor populations to resolve binding dynamics of alternative splicing regulators and neuronal RBPs during lineage commitment, demonstrating stage-specific shifts in exon recognition and transcript stability programs [131]. In spermatogenesis, profiling of enriched spermatogonial fractions has uncovered binding signatures of SRSF1, SRSF2, SRSF10, RBM46, and CWF19L2, implicating splicing control and RNA turnover in meiotic progression and germ cell differentiation [132,133,134,135,136,137]. These findings underscore how nucleotide-level RBP maps can illuminate stage-specific gene expression programs in tissues characterized by rapid transcriptional remodeling.

Beyond lineage-restricted systems, LACE-seq has also been applied in TNBC to define the RNA-binding landscape of SORBS2, linking its direct transcript interactions to lung metastasis-associated regulatory pathways [138]. Such applications illustrate that the advantages of reduced input and quantitative amplification are not limited to rare cell types but are also relevant in heterogeneous tumor environments where subpopulation-specific RBP activity may drive disease progression.

Another emerging strength of LACE-seq is its compatibility with integrative multi-omic strategies. Coupling LACE-seq with ribosome profiling or RNA modification mapping has enabled correlation of RBP binding sites with translational efficiency and epitranscriptomic marks, providing mechanistic insight into how binding position influences RNA fate [139]. These integrative approaches move beyond cataloging interaction sites and toward reconstructing functional regulatory circuits.

Despite its advantages, LACE-seq shares certain constraints inherent to UV crosslinking-based approaches. Crosslink efficiency remains protein- and sequence-dependent, and RT truncation signatures must be interpreted carefully to distinguish true contact sites from secondary structural barriers [125]. Nonetheless, by combining low-input sensitivity with nucleotide resolution and reduced amplification bias, LACE-seq represents a significant refinement within the CLIP family, expanding high-resolution RBP mapping into cellular contexts that were previously experimentally inaccessible.

Collectively, LACE-seq broadens the applicability of crosslinking-based RPI analysis across developmental, neuronal, reproductive, and disease-related systems. Its ability to interrogate scarce material without sacrificing precision positions it as a bridge between mechanistic biochemistry and physiologically relevant cellular models, particularly in fields where cell numbers and sample integrity are limiting factors.

While UV-crosslinking-based platforms such as CLIP and LACE-seq provide high-resolution maps of direct RNA–protein contacts, their dependence on UV irradiation, enzymatic trimming, and multiple biochemical purification steps can impose practical constraints [20,78,111,125]. Crosslinking efficiency varies across RNA sequence contexts and protein domains, and reverse transcription truncation events may reflect both *bona fide* contact sites and structural impediments [118]. Additionally, classical immunoprecipitation workflows introduce sample handling variability and material loss, which are particularly problematic in low-input or heterogeneous samples. These technical limitations have driven the development of methods that profile RNA–protein associations within intact nuclei while minimizing extensive biochemical manipulation.

Reverse Transcription-Based RPI Mapping

Assay of reverse transcription-based RBP Binding Site Sequencing (ARTR-seq) was developed to broaden the RNA classes accessible to reverse transcription–coupled RPI mapping [140]. In contrast to oligo(dT)-dependent approaches, ARTR-seq employs random-sequence primers during in situ reverse transcription, thereby enabling capture of both polyadenylated and non-polyadenylated transcripts [140]. This design is particularly important for RNA species such as histone mRNAs, numerous long noncoding RNAs, circular RNAs, and viral RNAs that lack canonical poly(A) tails yet play central regulatory roles. The workflow preserves spatial organization by performing reverse transcription within intact nuclei or permeabilized cells, followed by selective enrichment of cDNA fragments incorporating biotinylated nucleotides. This strategy enables mapping of RBP–RNA associations in both nuclear and cytoplasmic compartments without requiring UV crosslinking. Importantly, ARTR-seq maintains compatibility with extremely limited input material, including small cell populations and tissue cryosections [140]. Applications of ARTR-seq have demonstrated its versatility across regulatory contexts. Profiling of splicing regulators such as PTBP1 and RBFOX2 revealed transcript-specific binding patterns that align with alternative exon inclusion programs in differentiated neuronal populations [140]. In parallel, mapping of m6A reader proteins including YTHDF1, YTHDF2, and YTHDC1 showed compartment-specific RNA targeting consistent with their distinct roles in nuclear export, translation, and decay [140]. These findings illustrate how ARTR-seq can resolve functional partitioning of RBPs across subcellular domains. ARTR-seq has also enabled interrogation of nuclear architecture. Mapping transcripts enriched in nuclear speckles revealed preferential association of intron-retaining RNAs and splicing intermediates, suggesting a role for these compartments in post-transcriptional quality control [68,141]. By combining transcriptome-wide mapping with spatial context, ARTR-seq supports a systems-level understanding of how nuclear organization shapes RNA fate.

Reverse Transcribe and Tagment (RT&Tag) represents a conceptually related but mechanistically distinct approach [142,143,144]. Drawing inspiration from CUT&Tag chromatin profiling, RT&Tag eliminates UV crosslinking and instead recruits reverse transcription primers and a protein A–Tn5 fusion directly to antibody-bound proteins within intact nuclei [142,143]. Following primer annealing to polyadenylated RNA, reverse transcription and Tn5-mediated adapter insertion occur in situ, generating sequencing-ready cDNA fragments without classical immunoprecipitation. Because tagmentation is spatially confined to antibody-bound regions, RT&Tag enriches for RNAs associated with specific chromatin proteins while preserving native nuclear organization. This configuration allows interrogation of chromatin-associated RNAs and nascent transcripts linked to defined histone modifications or transcription factors. For example, RT&Tag has been used to capture RNAs associated with repressive histone marks, illuminating how transcriptional repression interfaces with RNA processing within intact nuclei [142].

Extensions of RT&Tag have broadened its temporal and epitranscriptomic reach. Integration with metabolic RNA labeling strategies enables time-resolved tracking of RNA synthesis and degradation within defined chromatin contexts [145,146,147]. Additionally, antibody-directed capture of m6A-modified transcripts via RT&Tag has linked epitranscriptomic marks to local chromatin environments, suggesting coordinated regulation between RNA modification and transcriptional state [68].

Compared to CLIP-derived approaches, ARTR-seq and RT&Tag reduce dependence on UV crosslinking and extensive post-lysis purification, offering streamlined workflows compatible with low-input and spatially resolved samples. However, because they do not rely on covalent crosslink stabilization in the same manner as UV-CLIP, careful interpretation and appropriate controls remain essential to distinguish direct binding from proximal co-residence.

Antibody dependence remains a shared limitation [68,140]. Variability in antibody specificity or epitope accessibility can affect enrichment profiles. To address this, CRISPR/Cas9-mediated epitope tagging of endogenous loci has become increasingly common, improving reproducibility across experiments and laboratories [148,149,150]. Emerging nanobody-based strategies further enhance targeting specificity while minimizing steric interference in nuclear environments [151].

Together, ARTR-seq and RT&Tag expand the methodological landscape beyond crosslinking-centric paradigms, providing complementary platforms that integrate RPI mapping with chromatin context, RNA modification profiling, and spatial resolution. Their adaptability across developmental, neuronal, and disease-related systems positions them as important tools for interrogating layered RNA regulatory networks in physiologically relevant settings.

##### RNA Tagging Platforms

Recent advances have shifted the conceptual framework of RPI studies from ex vivo biochemical capture of complexes toward direct, intracellular molecular recording of interaction events in living systems. Rather than isolating RNA–protein assemblies through crosslinking and immunoprecipitation, these approaches encode evidence of spatial proximity into the RNA or associated biomolecules themselves, preserving temporal and contextual information that may be lost during extraction [68,90]. Such strategies are particularly advantageous for detecting weak, transient, or rapidly remodeled RNA–protein associations that are difficult to stabilize using UV-dependent methodologies [68,152].

TRIBE-Based Approaches

Among the most conceptually distinct of these systems is TRIBE, which transforms RBP–RNA encounters into stable sequence-level marks [94]. Instead of capturing complexes biochemically, TRIBE fuses the RBP of interest to the catalytic domain of ADAR, allowing RNA editing to occur when the fusion protein contacts a transcript [94]. The resulting A→I (read as A→G) substitutions act as durable molecular signatures that can be detected by sequencing, effectively converting transient physical interactions into a heritable RNA footprint [94]. HyperTRIBE enhanced this framework by incorporating a hyperactive ADAR catalytic variant, such as the E488Q mutant, thereby substantially increasing editing frequency and reducing dependence on stringent local sequence constraints [153,154,155]. This increased sensitivity has proven particularly useful for RBPs with low-affinity or short-lived RNA interactions, although expression tuning remains essential to minimize nonspecific editing events [153,156]. Unlike CLIP-derived methods, which often require large cell numbers and multiple purification steps, TRIBE operates entirely within living cells, making it well suited to small or heterogeneous populations [94,153]. The biological applications of TRIBE have broadened considerably beyond early neuronal studies. While initial experiments in Drosophila neurons mapped Hrp48 and FMRP binding landscapes associated with synaptic development, subsequent adaptations have been implemented in mammalian stem cells, immune cells, and stress-responsive systems [94,153].

STAMP-Based Approaches

Beyond ADAR-based recording, complementary enzymatic strategies have expanded the mutational palette available for RPI mapping. Cytidine deaminase-based systems such as Surveying Targets by APOBEC-Mediated Profiling (STAMP) introduce C→U conversions, enabling detection of RBP proximity in sequence contexts less favorable for ADAR editing [157,158]. In STAMP, the RBP of interest is fused to an APOBEC1-derived cytidine deaminase, and C→U editing events introduced near binding sites are identified by RNA sequencing after subtraction of appropriate deaminase-only controls [157,158,159]. Unlike UV-based crosslinking methods, this approach encodes interaction information directly into the RNA molecule, allowing binding landscapes to be reconstructed without immunoprecipitation or complex biochemical recovery [157]. STAMP has been successfully applied to profile canonical RBPs such as RBFOX2 and TIA1, where editing signatures were enriched near known binding motifs, validating the approach against established CLIP datasets [157]. In addition, fusion of APOBEC1 to ribosomal proteins (ribosome-STAMP) enabled identification of actively translated mRNAs, linking proximity editing to translational engagement and ribosome-associated RNA pools [157]. These studies demonstrate that STAMP can capture both sequence-specific RBP targets and broader ribonucleoprotein environments under physiological conditions.

A defining strength of STAMP is its compatibility with low-input and single-cell workflows. Brannan et al. demonstrated that STAMP-derived editing signatures can be resolved at single-cell resolution, enabling cell-type-specific RBP target discovery in heterogeneous populations. This expands the method’s applicability to developmental systems, tumor microenvironments, and immune cell subsets where bulk approaches obscure cell-state-specific binding programs. Moreover, because APOBEC-mediated editing is not constrained by the structural requirements of ADAR, STAMP can reveal RNA targets lacking suitable adenosines for TRIBE-based recording, thereby complementing A→I editing platforms [157].

However, interpretation of STAMP data requires attention to biochemical and structural constraints. Cytidine editing depends on local RNA accessibility and sequence context, and editing efficiency reflects proximity and dwell time rather than direct base-contact affinity [157,158]. False negatives may occur if editable cytidines are absent near true binding sites, whereas background editing can arise from transient spatial proximity or elevated fusion expression. Accordingly, best practice includes deaminase-only controls, expression titration, replicate concordance thresholds, and orthogonal validation using CLIP or reporter assays [157,158]. More recently, dual-deaminase recording strategies combining A→I and C→U conversion modules have been proposed to multiplex RBP proximity mapping within the same cellular system, providing higher-resolution dissection of overlapping RNA regulatory networks. Together, these innovations position STAMP as a powerful and complementary tool within the broader class of editing-based molecular recording approaches for RPI analysis. STAMP has also been applied to delineate genome-wide RNA association patterns of poly(A)-binding proteins located in distinct cellular compartments, specifically PABPN in the nucleus and PABPC in the cytoplasm [160]. These analyses revealed that RNA molecules display compartment-specific engagement with these proteins, with binding preferences influenced by factors such as splicing completion, poly(A)-tail length, transcript stability, and translational activity. Such observations underscore the dynamic redistribution of poly(A)-binding proteins during RNA maturation and export, highlighting how nuclear and cytoplasmic PABPs differentially regulate RNA fate [160].

An additional innovation involved combining STAMP with TRIBE-based RNA editing, generating a dual-recording system referred to as TRIBE-STAMP. In this configuration, distinct RBPs are fused separately to APOBEC1 and ADAR catalytic domains, enabling simultaneous cytidine and adenosine editing marks on shared RNA substrates [161]. This strategy demonstrated that individual transcripts can be targeted either sequentially or concurrently by multiple YTHDF family proteins throughout their lifecycle [161]. By enabling detection of overlapping RBP occupancy on single RNA molecules, TRIBE-STAMP provides a framework for investigating cooperative, competitive, and temporally coordinated RBP interactions. This multiplexed molecular recording approach significantly enhances the ability to resolve combinatorial regulatory logic governing RNA stability, translation, and decay [162].

APEX-Based RNA Proximity Labeling

Parallel to editing-based systems, enzymatic proximity-labeling approaches such as APEX-seq, as illustrated in Figure 4B, which schematically represents proximity-based RNA labeling and downstream identification workflows, have provided a powerful framework for interrogating RNA localization and RPI landscapes with spatial precision in living cells [24,163,164]. APEX-seq builds on the catalytic activity of the engineered peroxidase APEX2, which generates short-lived biotin–phenoxyl radicals that covalently label nearby RNA molecules within a restricted radius (~10–20 nm), thereby capturing RNAs that reside within defined molecular microenvironments [24,42,163]. By targeting APEX2 to defined subcellular structures—such as the mitochondrial matrix, outer mitochondrial membrane (OMM), nuclear lamina, nucleolus, and nuclear pore complex—APEX-seq enables transcriptome-wide profiling of RNAs residing within these compartments under near-native conditions [24].

Importantly, APEX-seq has also been applied in a protein-centric configuration, where APEX2 is fused directly to RNA-binding or translation-associated proteins to resolve RNA–protein assemblies in vivo. For example, APEX2 was fused to the DEAD-box helicase eIF4A1 and the cap-binding protein eIF4E1; core components of the eIF4F translation initiation complex to map RNAs positioned near active translation machinery [164]. In these experiments, APEX2-eIF4A1 and APEX2-eIF4E1 selectively enriched mRNAs encoding translation-related proteins and revealed distinct spatial distributions within the 43S preinitiation complex, demonstrating that APEX-seq can resolve positional organization within RNP assemblies rather than merely global localization [164].

APEX-seq has further been used to examine stress-induced RNA–protein condensates. When APEX2-eIF4A1 was profiled during heat shock or hippuristanol treatment, distinct RNA populations were enriched in stress granules, and the RNA composition differed depending on the stressor, indicating that RNP remodeling is context dependent [164]. These studies showed that transcripts with longer open reading frames accumulated in stress granules at later time points, linking translation elongation dynamics to RNP assembly kinetics [164]. Such findings illustrate that APEX-seq can capture dynamic RNA–protein networks reorganized by cellular stress, which are often difficult to stabilize using crosslinking-based approaches. Mechanistically, APEX-based RNA labeling differs from crosslink-dependent methods in that RNA molecules are directly biotinylated without requiring RNA–protein crosslinking, eliminating additional fixation steps that can degrade spatial specificity [163]. Because labeling occurs within approximately one minute, APEX-seq enables temporal interrogation of RNA localization and RNP assembly during perturbations such as mTOR inhibition or stress induction [24,164].

Beyond RNA-focused profiling, APEX chemistry has also been adapted to Chromatin Identification (ChromID), in which proximity labeling is directed to specific chromatin marks via engineered chromatin readers fused to biotin ligases such as BASU [109,163,165]. While ChromID primarily identifies protein interactomes at defined chromatin modifications, it conceptually extends APEX-style spatial labeling into DNA-associated regulatory complexes, highlighting how proximity chemistry can bridge RNA–protein and chromatin-centered regulatory networks [163]. Collectively, APEX-seq and related proximity-labeling platforms extend RPI studies beyond binding-site resolution to include spatial organization and compartment-specific regulation. By integrating RNA labeling with proteomic proximity mapping, these methods provide insight into how translational complexes (e.g., eIF4E1, eIF4A1), stress granule scaffolds (e.g., G3BP1, TIA1), and organelle-associated RNPs coordinate RNA fate within intact cellular environments [24,72,166,167].

PUP-Based RNA Tagging

An alternative strategy for in vivo RPI profiling relies on enzymatic RNA tailing rather than nucleotide editing or biotin-based proximity labeling. Poly(U) polymerase (PUP)-based RNA tagging exploits the catalytic activity of terminal uridylyl transferases to append non-templated uridine residues to RNA 3′ ends when positioned near an RBP of interest [168,169]. Instead of marking RNA through base substitution (as in TRIBE) or radical-mediated biotinylation (as in APEX-seq), this system encodes RBP proximity as a covalent uridine “tail” directly appended to the target transcript.

In the canonical implementation, the RBP is fused to a poly(U) polymerase, typically derived from *C. elegans* PUP-2, which lacks strong intrinsic RNA-binding specificity but retains catalytic uridylation activity [169]. When the fusion protein engages an RNA in vivo, uridines are added to its 3′ terminus. These artificial U-tails are subsequently detected through specialized reverse transcription and sequencing workflows that enrich for uridylated transcripts [169]. Because uridylation occurs at the RNA terminus, it provides a distinct biochemical signature that can be distinguished from endogenous polyadenylation or RNA decay intermediates.

One of the earliest applications of PUP-based tagging was the mapping of targets of the PUF family RBP Puf3p in yeast, where the approach revealed enrichment of mRNAs encoding mitochondrial proteins, consistent with Puf3p’s established role in mitochondrial biogenesis regulation [169]. In this study, uridine tail length and frequency correlated with RBP occupancy, allowing discrimination between strongly and weakly associated transcripts.

Subsequent implementations extended PUP tagging to additional RBPs such as Bfr1p and Khd1p, enabling comparative profiling of RNA-binding specificity within overlapping regulatory networks in budding yeast [169,170]. These analyses demonstrated that distinct RBPs can produce differential uridylation patterns on shared transcript pools, highlighting the potential of the method to resolve combinatorial RNA regulation. The versatility of RNA tagging has facilitated its deployment in organisms ranging from yeast to human cells, underscoring its adaptability across evolutionary and cellular complexity [169,171,172].

Unlike crosslink-dependent methods, PUP-based tagging does not require UV irradiation or chemical fixation, thereby avoiding crosslinking bias and enabling analysis under physiological growth conditions [169]. However, because uridylation occurs at RNA 3′ ends, transcripts undergoing rapid decay or alternative 3′ processing may be differentially represented. Importantly, the quantitative dimension of PUP tagging reflected in U-tail length and abundance has been leveraged to infer relative binding strength or dwell time, although this interpretation must account for RNA stability and turnover kinetics [169].

From a mechanistic standpoint, PUP-based RNA tagging differs from editing-based systems such as TRIBE in that it records interactions at transcript termini rather than near internal binding sites. This distinction reduces dependence on internal sequence context (e.g., editable adenosines or cytidines) but introduces sensitivity to transcript 3′-end accessibility and poly(A)-binding protein occupancy [169]. Recent refinements have incorporated inducible expression systems and temporal control to restrict uridylation windows, thereby improving temporal resolution of RNA–protein association dynamics. Collectively, PUP-mediated RNA tagging offers a complementary approach within the broader class of enzymatic RPI recording strategies. By converting RBP proximity into terminal uridine extensions rather than base substitutions or radical tags, it provides a mechanistically distinct and minimally perturbative means to profile RPI landscapes in living cells.

Emerging and High-Throughput Extensions

High-throughput and microfluidic adaptations have further extended proximity recording into single-cell and subcellular domains. Particle-templated partition sequencing (PIP-seq) integrates droplet-based workflows with proximity-tagging chemistry, enabling resolution of RNA–protein networks in complex tissues where cellular heterogeneity would otherwise obscure interaction landscapes [173,174,175]. Such tools are particularly valuable in neurodegenerative disease models and tumor microenvironments, where rare cell states may drive pathology [174].

Although editing-based proximity systems offer major advantages, interpretation requires caution (Table 5). Editing events represent proximity rather than stoichiometric binding strength; frequency is influenced by RNA abundance, structural accessibility, dwell time, and enzyme expression level [153,156]. In addition, endogenous ADAR activity can introduce background edits in certain tissues, necessitating rigorous controls and replicate concordance [176]. Computational analysis typically involves strand-aware alignment, variant filtering, and aggregation of clustered edits into candidate loci prior to orthogonal validation using CLIP, reporter assays, or mutational analysis [153,177].

Recent innovations aim to introduce temporal precision and spatial confinement into editing-based RPI mapping (Table 5). Split-enzyme and optogenetically inducible variants permit pulse labeling of RBP interactions in response to defined stimuli. Light-activated ADAR fusions have been shown to restrict editing to subcellular regions exposed to patterned illumination, enabling spatially restricted RNA–protein recording in live cells. Targeting adaptors such as dCas13 further refine editing to defined RNA neighborhoods, integrating programmable RNA targeting with proximity recording [178].

Collectively, molecular recording strategies such as TRIBE, HyperTRIBE, STAMP-like systems, and APEX-seq represent a conceptual evolution in RPI analysis. By embedding interaction signatures directly within RNA molecules, these approaches circumvent many of the biochemical constraints of immunoprecipitation-based methods and enable dynamic, context-aware profiling of RNA–protein networks in living cells [68,90]. As mutational chemistries, temporal control modules, and single-cell sequencing platforms continue to advance, RNA editing-based recording systems are poised to deepen our understanding of how RPIs orchestrate cellular function and contribute to disease.

### 2.3. Image-Based Methods

Image-based approaches for studying RPIs are founded on the direct visualization of RNA molecules and their associated proteins within intact cellular environments, allowing interrogation of interactions in their native spatial context. Unlike biochemical enrichment or sequencing-based strategies, imaging techniques preserve cellular architecture and enable assessment of where RPIs occur relative to subcellular compartments and structural landmarks such as stress granules, processing bodies, nuclear speckles, and chromatin-associated domains [179,180,181,182]. These approaches are particularly valuable for resolving cell-to-cell heterogeneity and for identifying compartment-specific interactions that may be obscured in bulk biochemical assays [183,184].

At the core of image-based RPI detection is the principle of spatial proximity, whereby RNA molecules and proteins are simultaneously visualized and assessed for co-localization within a defined optical or molecular distance. RNA detection is most commonly achieved using fluorescence in situ hybridization (FISH) or engineered RNA-tagging systems, while proteins are visualized through immunofluorescence or genetically encoded fluorescent reporters [183,184]. The extent of spatial overlap between RNA and protein signals provides an initial indication of potential association, which can be further refined using proximity-dependent signal amplification strategies [185]. Importantly, imaging approaches enable discrimination between diffuse cellular co-distribution and interactions confined to discrete functional microenvironments [183,186].

A key strength of image-based RPI methods lies in their ability to reveal interaction context rather than interaction abundance. While most imaging approaches do not directly quantify binding affinity, they excel at resolving interaction localization, temporal dynamics, and structural organization of RNP assemblies [183]. Live-cell imaging platforms further extend this capability by enabling real-time observation of RPI dynamics, including RNA transport, remodeling of RNPs, and stimulus-dependent recruitment or release of RBPs [186,187]. Such dynamic information is essential for understanding transient or regulated interactions that are difficult to capture using fixation-based or crosslink-dependent techniques [183].

Image-based RPI detection strategies differ in their interaction specificity, which is determined by both spatial resolution and the molecular mechanism underlying signal generation. Conventional diffraction-limited fluorescence microscopy infers interactions primarily through co-localization, which does not necessarily reflect direct physical contact between RNA and protein [188]. To increase specificity, techniques such as RNA-PLA or Förster resonance energy transfer (FRET) restrict signal generation to nanometer-scale distances, thereby strengthening inference of near-direct molecular association [22,189]. Super-resolution microscopy further enhances spatial precision by resolving RNA–protein assemblies below the diffraction limit, providing insights into the nanoscale organization and architecture of RNP complexes [190,191].

Despite their advantages, image-based approaches are subject to important technical and interpretive considerations. Fluorescent labeling strategies may perturb RNA structure or protein function, fixation procedures can alter molecular distributions, and apparent signal overlap may arise from high local concentrations rather than true interaction [185,189]. Consequently, image-based RPI detection methods are most powerful when integrated with orthogonal biochemical or sequencing-based approaches, either to validate molecular interactions or to contextualize them within native spatial and temporal frameworks. As imaging technologies continue to advance through improved probes, higher resolution modalities, and integration with spatial and single-cell omics, image-based approaches are increasingly positioned as a critical component of comprehensive RPI analysis [192,193].

#### 2.3.1. Fixed-Cell Imaging Approaches

Fixed-cell imaging approaches provide a central framework for studying RPIs by preserving cellular architecture and enabling spatial interrogation of RNA molecules together with their associated proteins at endogenous abundance [28,193]. Chemical fixation stabilizes subcellular organization, allowing RNA–protein associations to be examined within defined compartments, where biochemical fractionation often loses spatial information [183]. A major advantage of these assays is their broad applicability to diverse cell types and tissues, including clinical samples, because they do not require genetic engineering or exogenous enzyme expression [194]. However, fixation can influence macromolecular organization and accessibility of epitopes or RNA sequences, so conclusions about molecular association are strongest when imaging readouts are interpreted alongside orthogonal biochemical or crosslinking-based evidence [195].

**Figure 5:**
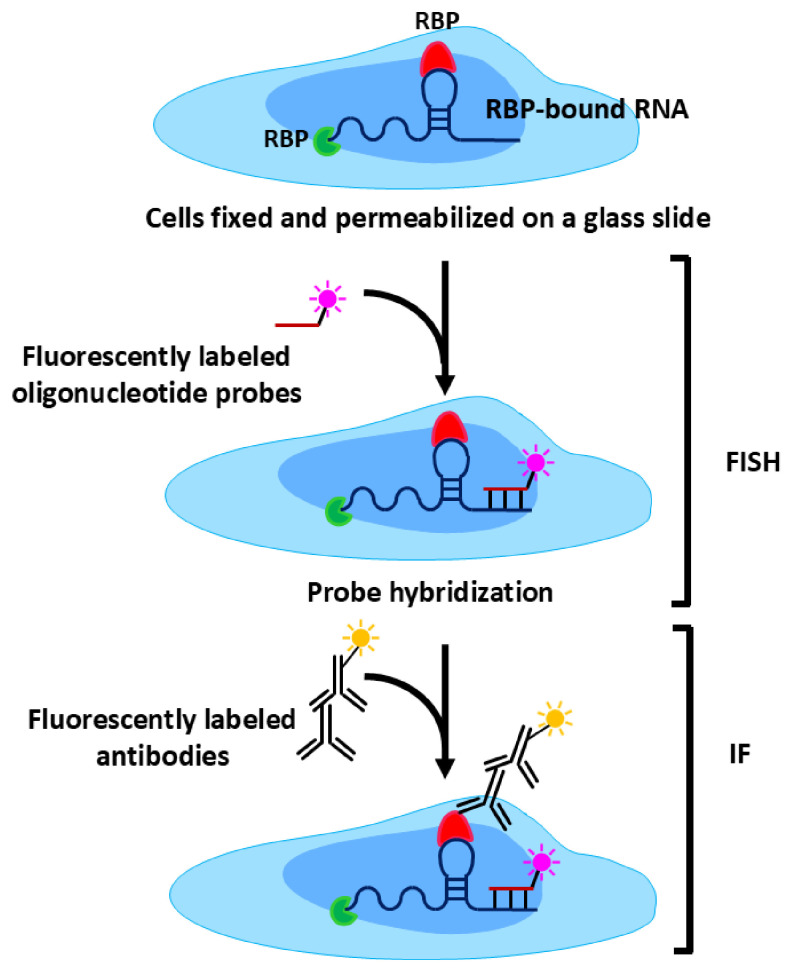
Schematic illustration of RNA-FISH combined with immunofluorescence (RNA-FISH/IF). Target RNAs are detected in fixed and permeabilized cells by hybridization of fluorescent RNA-FISH probes, followed by immunofluorescence staining of the corresponding RNA-binding protein (RBP, represented by pink and green icons) using specific antibodies. Colocalization of RNA and protein fluorescence signals is assessed by fluorescence microscopy, enabling spatial visualization of RNA–protein associations at subcellular resolution.

RNA fluorescence in situ hybridization (RNA FISH) is a cornerstone technique for visualizing RNA molecules in fixed cells and has been widely applied to study RNA localization and RNA–protein co-distribution [183,196,197]. In this approach, cells are first fixed and permeabilized on a glass slide to preserve RNA–protein complexes while allowing probe access. Classical RNA FISH relies on fluorescently labeled oligonucleotide probes that hybridize to complementary RNA sequences, enabling detection of specific transcripts within intact cellular contexts [183,197]. As illustrated in Figure 5, RNA FISH can be combined with immunofluorescence (IF) using fluorescently labeled antibodies targeting RBPs, allowing simultaneous visualization of RNA molecules and their associated proteins within the same cell. The development of single-molecule RNA FISH (smFISH), which employs multiple short, singly labeled oligonucleotide probes that tile across a target RNA, dramatically improved sensitivity and specificity, allowing individual RNA molecules to be detected and quantified at single-cell resolution and has become a foundational method for transcript-resolved RPI analysis [198].

In typical smFISH implementations, multiple probes collectively bind a target RNA, enabling robust signal generation and improving specificity through multiple hybridization events rather than reliance on single-probe detection [198,199]. Building on the general RNA FISH–IF framework (Figure 5), smFISH combined with IF provides single-molecule resolution, enabling quantitative spatial analyses of RPIs. This approach allows precise assessment of RBP enrichment at individual transcript sites, as well as compartment-specific RNA localization patterns [183,193]. Because diffraction-limited co-localization operates at the scale of the microscope point-spread function (typically ~200–300 nm laterally), signal overlap in conventional imaging is most appropriately interpreted as shared occupancy within an optical volume rather than definitive evidence of direct physical contact [200]. This is particularly relevant in high-density nuclear domains where both RNA and protein signals can accumulate due to compartmental partitioning even without direct binding.

Recent methodological advances have substantially expanded RNA FISH from single-transcript visualization to high-content spatial profiling approaches that enable RPI analyses within broader cellular and transcriptomic contexts. Highly multiplexed FISH strategies, including Multiplexed Error-Robust Fluorescence In Situ Hybridization (MERFISH) and seqFISH, now allow quantification of hundreds to thousands of RNA species per cell, facilitating parallel assessment of RNA–protein co-distribution across multiple RNA classes and enabling identification of compartment-specific RNA programs that recruit distinct repertoires of RBPs [201,202]. Integration of FISH with super-resolution microscopy further refines spatial resolution beyond the diffraction limit, permitting nanoscale analysis of RNP organization and RNA substructure within nuclear bodies and cytoplasmic granules at tens-of-nanometers scale [182,203]. These advances have proven particularly informative for spatially organized RNP systems, such as β-actin mRNA localization networks, where smFISH combined with immunofluorescence has revealed enrichment of zipcode-binding RBPs, including ZBP1 (IGF2BP1), at localized transcripts in protrusive regions of migrating cells, linking RNA positioning to translational regulation [183,204]. In the nucleus, RNA FISH has enabled detailed mapping of lncRNAs relative to protein-rich compartments, exemplified by MALAT1 co-localization with serine/arginine-rich splicing factors such as SRSF1 and SRSF2 within nuclear speckles, supporting a scaffolding role for lncRNAs in organizing splicing-associated RNP environments [205]. Similarly, XIST RNA localization on the inactive X chromosome has been visualized by FISH in conjunction with immunofluorescence for chromatin-associated proteins including HNRNPU (SAF-A), SPEN, and Polycomb group components, enabling spatial correlation between RNA coating and protein recruitment during X-chromosome inactivation [206,207].

**Figure 6:**
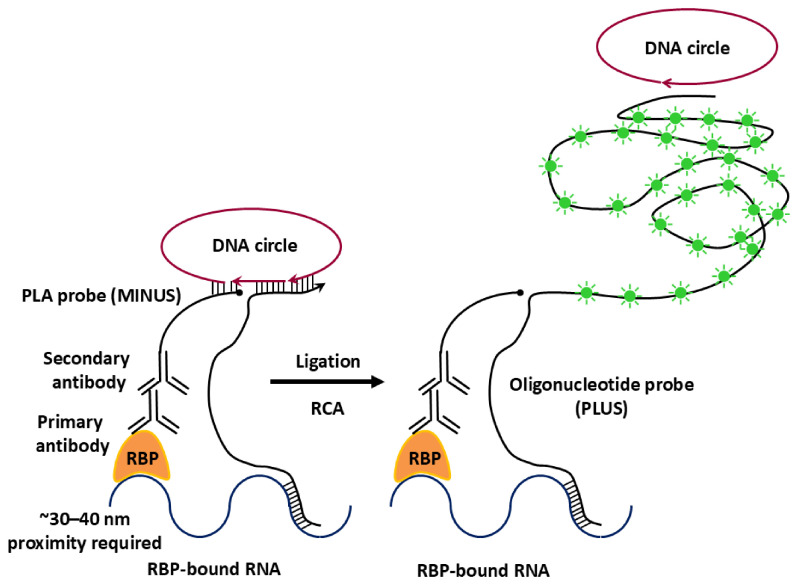
Schematic illustration of RNA-PLA. RBPs (represented by orange icons) associate with target RNAs in fixed cells and are detected using protein-specific antibodies and RNA-targeting probes. When the RNA and RBP are in close spatial proximity (typically ~30–40 nm), PLA probes enable ligation and rolling-circle amplification, generating a localized amplified signal that appears as discrete fluorescent puncta. Accumulation of these signals reflects RNA–protein proximity events and is visualized by fluorescence microscopy.

To move beyond co-distribution and strengthen inference of molecular association in fixed samples, RNA-PLA adapts proximity ligation chemistry to report RNA–protein proximity events through localized signal amplification [22,208]. RNA-PLA generates fluorescence only when RNA- and protein-targeted probes are sufficiently close to enable ligation and subsequent rolling-circle amplification, producing bright puncta that represent discrete proximity events rather than diffuse overlap (Figure 6) [22]. In the RNA-PLA workflow, the target RNA is first hybridized with a single-stranded DNA oligonucleotide (“PLUS probe”) containing a 40–50 nucleotide antisense region complementary to the RNA, while the protein is simultaneously recognized by a primary antibody and a secondary antibody conjugated to a complementary DNA oligonucleotide (“MINUS probe”), enabling spatially constrained probe pairing only when the RNA and protein reside within the same molecular complex as illustrated in Figure 6 [22,208]. This approach effectively shifts the detection criterion from diffraction-limited co-localization to a nanometer-scale proximity threshold, and classical PLA characterization places the effective interaction radius at approximately ~30–40 nm, which is substantially more stringent than conventional co-localization but still broader than direct-contact readouts such as FRET (<~10 nm) [209]. This proximity constraint also has important implications for probe design, as the RNA-targeting oligonucleotide should be selected to maximize the likelihood that the probe and antibody-conjugated oligonucleotides fall within the required ~30–40 nm interaction radius. In practice, probe placement is guided by RNA accessibility and, where possible, proximity to known or predicted RBP-binding regions, while the probe sequence must be carefully designed to ensure high hybridization specificity and minimize off-target binding. Because RNA-PLA relies on hybridization to accessible regions of the transcript, poorly positioned or nonspecific probes may reduce signal efficiency or increase background, underscoring the importance of optimizing both probe sequence and binding location, typically using antisense oligonucleotides selected from accessible regions of the target RNA [208]. RNA-PLA signal generation further requires enzymatic ligation of a circular DNA template followed by rolling-circle amplification (RCA), producing a localized amplicon that can be detected by fluorescently labeled complementary oligonucleotides as depicted in Figure 6, thereby providing high sensitivity even for low-abundance or transient RNA–protein complexes [22]. Importantly, experimental validation using endogenous systems has demonstrated that RNA-PLA signals depend on the presence of a *bona fide* protein-binding site on the target RNA, as loss or mutation of the binding motif substantially reduces or abolishes the proximity signal, underscoring the assay’s specificity for biologically relevant RPIs.

Because RNA-PLA is compatible with endogenous targets and tissue sections, it has been used as an in situ validation strategy for candidate RPIs involving mRNAs and lncRNAs, particularly in nuclear contexts where chromatin-associated assemblies and compartmental crowding complicate interpretation of standard co-localization [210]. For example, RNA-PLA has confirmed close proximity between MALAT1 and lncRNA, providing stronger evidence for molecular association than co-localization alone [210]. RNA-PLA has also been applied to visualize interactions between viral RNAs and host RBPs during infection, enabling in situ validation of virus–host RNA–protein interfaces involving factors implicated in RNA processing, export, and replication [211].

Methodological refinements continue to increase the utility of fixed-cell imaging for RPI studies. For FISH, improvements in probe design algorithms, error-robust barcoding, and amplification chemistries have increased detection efficiency for low-copy RNAs while supporting multiplexed readouts suitable for spatially resolved RPI hypothesis generation at scale [22,204,205]. For RNA-PLA, advances in probe conjugation chemistry, optimized amplification strategies, and integration with quantitative image analysis pipelines have improved sensitivity and reduced nonspecific background, supporting more reliable comparative analyses across conditions [22]. Nonetheless, RNA-PLA, like other proximity-based approaches, can report near-neighbor association within the ligation radius and therefore may capture indirect interactions within larger assemblies, reinforcing the need for rigorous negative controls (e.g., RNase treatment, probe omission, non-targeting antibodies) and orthogonal validation such as CLIP or RNA pulldown for mechanistic conclusions [20,22,78]. Collectively, fixed-cell imaging approaches provide a scalable and spatially explicit layer of evidence for RPIs, enabling linkage of RNA–protein association patterns to subcellular organization, cellular heterogeneity, and condition-specific remodeling of RNP architectures [22,183,203,204,205,206,207].

Beyond RNA FISH and RNA–PLA, several additional fixed-cell imaging approaches contribute important conceptual and spatial perspectives to RPI analysis by exploiting different physical principles of molecular proximity and resolution. FRET-based imaging (less common in fixed cells) relies on non-radiative energy transfer between donor and acceptor fluorophores and is therefore sensitive to very short intermolecular distances, typically below ~10 nm, making it one of the most stringent optical methods for inferring direct RPIs, albeit usually in simplified or engineered systems [209]. Super-resolution fluorescence microscopy techniques such as Stochastic Optical Reconstruction Microscopy (STORM), Photoactivated Localization Microscopy (PALM), Single-Molecule Localization Microscopy (SMLM) and Structured Illumination Microscopy (SIM) circumvent the diffraction limit of conventional light microscopy to achieve spatial resolutions on the order of ~20–50 nm, enabling visualization of the nanoscale organization of RNP assemblies and discrimination of closely spaced RNA–protein structures that appear co-localized in standard confocal imaging [182,203]. Expansion Microscopy (ExM) takes a complementary physical approach by isotropically enlarging fixed samples, commonly by four- to tenfold, thereby translating nanoscale RNA–protein spatial relationships into resolvable distances using conventional microscopes, and has been combined with RNA FISH and immunostaining to examine RNA–protein organization in dense nuclear or cytoplasmic environments [212,213]. Collectively, these approaches leverage distinct physical principles such as energy transfer, optical resolution enhancement, sample expansion, or electron scattering to probe RNA–protein proximity across different spatial scales, and they are most informative when used as targeted validation or contextualization tools alongside RNA-centric, protein-centric, and proximity-labeling strategies (Table 6).

#### 2.3.2. Live-Cell Imaging Approaches

Live-cell imaging approaches for studying RPIs encompass a diverse set of strategies that aim to visualize RNA molecules, RBPs, or their mutual associations within living cells while preserving native dynamics and cellular context. Unlike fixed-cell imaging, live-cell methods enable direct observation of interaction kinetics, spatial reorganization, and temporal regulation of RNP assemblies during processes such as transcription, RNA transport, translation, and stress responses [183,197,214,215]. Conceptually, live-cell RPI imaging approaches can be grouped into RNA-centric strategies that label the RNA itself, protein-centric strategies that track RNA-associated proteins, and proximity- or interaction-dependent optical readouts that report close RNA–protein association through fluorescence changes [215]. Among these, live-cell RNA tagging approaches represent the most widely established and experimentally accessible class, while complementary approaches such as CRISPR/Cas13-based RNA imaging, and FRET-based proximity sensing offer higher specificity or access to endogenous transcripts in selected contexts [215,216,217,218].

Live-cell RNA tagging approaches enable direct visualization of RNA molecules and their associated proteins in real time, providing a dynamic perspective on RPIs that cannot be captured using fixed-cell or biochemical methods. By preserving cellular viability, these techniques allow researchers to follow RNA localization, transport, and assembly into RNP complexes while capturing rapid changes in interaction state that occur during transcription, translation, and RNA trafficking [183,184,215]. This temporal dimension is particularly valuable for studying transient or regulated RNA–protein associations that form and dissolve on timescales ranging from seconds to minutes [215].

Most live-cell RNA tagging strategies rely on genetically encoded RNA labels that recruit fluorescently tagged RBPs to specific RNA molecules. The most widely used platforms are the MS2 and PP7 systems, in which arrays of bacteriophage-derived RNA stem loops are engineered into the RNA of interest and bound by their cognate coat proteins fused to fluorescent reporters [219,220]. Recruitment of multiple coat proteins to a single RNA amplifies fluorescence signal sufficiently to allow visualization of individual transcripts in living cells, with spatial resolution limited by diffraction (~200–300 nm) but temporal resolution determined by imaging speed [219,220].

Live-cell RNA tagging has been particularly informative for dissecting RPIs involved in mRNA localization and localized translation. Classic MS2-based studies of β-actin mRNA demonstrated dynamic recruitment of zipcode-binding proteins such as ZBP1 (IGF2BP1), revealing how reversible RPIs regulate transcript transport and translational repression during cell migration [220]. These experiments showed that β-actin mRNAs undergo cycles of RBP binding and release as they move along cytoskeletal tracks, highlighting the kinetic nature of RPI regulation that is inaccessible to fixed-cell methods [220].

Beyond cytoplasmic mRNAs, live-cell RNA tagging approaches have provided direct insight into RNA–protein interaction dynamics within intact cells. A seminal example is the real-time visualization of ZBP1 association with β-actin mRNA, in which fluorescently labeled ZBP1 and tagged β-actin transcripts were simultaneously tracked in living cells [221]. This dual-color strategy integrates RNA-centric tagging with protein-centric live-cell imaging, enabling simultaneous visualization of both the transcript and its cognate RBP within the same cellular context. This study demonstrated that ZBP1 binds β-actin mRNA cotranscriptionally in the nucleus and remains associated during nuclear export and subsequent cytoplasmic transport. The ZBP1–β-actin ribonucleoprotein complex exhibited directed cytoplasmic movement consistent with motor-dependent transport. Moreover, mislocalization of ZBP1 resulted in altered β-actin mRNA distribution, establishing a functional link between RBP binding and transcript localization. By capturing the coordinated trafficking of RNA and protein over time, this work provided early real-time evidence that RBP association is not static but dynamically maintained throughout multiple stages of RNA maturation, export, and intracellular targeting [221].

Protein-centric live-cell imaging approaches, in which candidate RBPs are fluorescently tagged and tracked in living cells, have also been used to examine RBP recruitment dynamics, residence times, and exchange kinetics within RNA-rich compartments or at active transcription sites, providing insight into how proteins engage with RNA populations in real time [221]. In contrast to RNA-centric tagging, these approaches do not directly visualize specific RNA molecules but instead infer RNA engagement from RBP behavior, such as accumulation at transcription foci, stress granules, or other RNP assemblies. Together, RNA-centric tagging and protein-centric tracking strategies provide complementary views of live RPI dynamics. One emphasizes transcript movement and RBP co-trafficking, and the other focuses on protein mobility and exchange within RNA-rich microenvironments.

Overall, live-cell RNA tagging approaches provide a unique window into the dynamic and reversible nature of RPIs by revealing when, where, and for how long specific RNAs associate with protein partners inside living cells (Table 7). Although these methods primarily infer interaction through spatial co-movement rather than direct molecular contact, their ability to capture temporal dynamics makes them an essential complement to fixed-cell imaging, proximity labeling, and RNA-centric biochemical approaches in comprehensive RPI analysis [183].

Live-cell FRET-based strategies offer a more stringent optical readout of RNA–protein proximity by reporting interactions occurring within ~1–10 nm, consistent with direct molecular contact, but they typically require engineered labeling schemes and are therefore limited to targeted, low-throughput applications [218]. CRISPR/Cas13-based live-cell RNA imaging has emerged as an alternative RNA-centric approach that enables visualization of endogenous RNAs without introducing large RNA tags, and has been adapted to study RNA localization and RNA–protein neighborhood associations in living cells, generally at diffraction-limited spatial resolution. The programmable nature of Cas13, guided by sequence-specific crRNAs, allows precise targeting of endogenous transcripts through direct RNA–RNA base pairing, eliminating the need for genomic integration or extensive RNA engineering. The reviewed applications highlight both catalytically active Cas13 variants for targeted RNA degradation and catalytically inactive (dCas13) forms for RNA binding without cleavage, underscoring its versatility as a modular RNA-targeting platform. In cancer research contexts, Cas13 systems have been applied to selectively silence oncogenic transcripts, modulate aberrant RNA expression profiles, and perform RNA editing, demonstrating high specificity and minimal off-target genomic effects. These programmable RNA-recognition capabilities provide a conceptual foundation for adapting Cas13 platforms to visualization-based applications, complementing traditional RNA tagging strategies in the broader study of RNA biology [217]. Collectively, these strategies complement live-cell RNA tagging approaches by offering alternative perspectives on RNA–protein association that emphasize protein dynamics, direct proximity, or endogenous RNA targeting.

## 3. Strategic Considerations in RNA–Protein Interaction Analysis

The diversity of RPIs described throughout this review reflects the multifaceted roles that RNAs play in gene regulation, ranging from stable structural scaffolding to highly dynamic regulatory interactions. Consequently, the choice of experimental approach should be guided not by methodological popularity, but by the specific biological question being addressed, including whether the focus is on interaction discovery, binding specificity, spatial organization, or temporal dynamics. Because each class of RPI detection method captures a distinct layer of information, deliberate method selection and integration remain central to obtaining biologically meaningful insight [12,25,28,124].

When the biological question is anchored to a specific RNA, RNA-centric approaches provide the most direct entry point. Hybridization-based pulldown strategies such as ChIRP, CHART, RAP-MS, iDRiP, and MORPH-MS are particularly effective for identifying protein assemblies formed on defined RNAs and have been widely applied to chromatin-associated lncRNAs. For example, RNA-centric pulldown approaches were instrumental in resolving the XIST–HNRNPU (SAF-A) and XIST–SPEN complexes, clarifying how XIST recruits chromatin modifiers to establish X-chromosome inactivation [31,32,33,34,35,36,37]. Similarly, RAP-MS and related methods identified MALAT1–SRSF1/SRSF2 interactions, supporting a role for MALAT1 in organizing splicing factor-rich nuclear speckles [34,35]. RNA-centric pulldown strategies have also been applied to viral RNAs, including influenza and HIV transcripts, uncovering host RBPs that regulate viral replication and immune evasion. These approaches are most appropriate when comprehensive discovery of RNA-associated proteins is required, but because they often capture both direct binders and indirect interactors within larger RNP assemblies, they should be complemented by methods that refine binding specificity.

RNA-centric proximity labeling approaches such as RaPID and RNA-BioID offer increased sensitivity for transient or spatially restricted RNA-associated proteins in living cells, and have been used to map dynamic protein neighborhoods around regulatory RNAs that are poorly captured by crosslinking-based pulldowns [28,43]. However, because these approaches report molecular proximity rather than direct contact, they are best suited for identifying candidate interactors that require subsequent validation. CRISPR/Cas13-assisted RNA-centric methods, including CARPID and CRUIS, are particularly advantageous when endogenous RNA context must be preserved, as demonstrated by mapping protein partners of low-abundance lncRNAs and circular RNAs in situ [40,46,48,84].

When the experimental emphasis shifts to protein-centric questions, the choice of method depends on whether intrinsic RNA-binding preferences or cellular interaction landscapes are of interest. In vitro approaches such as SEQRS and HTR-SELEX are well suited for defining the sequence and structural determinants of RNA recognition by RBPs under controlled conditions. These methods have clarified intrinsic binding preferences of proteins such as ZBP1 (IGF2BP1) and splicing regulators, providing mechanistic insight into why specific RNA motifs are recognized [19,103,104]. However, intrinsic specificity profiles often diverge from in vivo binding patterns due to RNA folding, competition among RBPs, and subcellular localization.

Protein-centric in vivo RNA-tagging approaches bridge this gap by capturing RNA targets of RBPs in their native cellular environment. TRIBE and HyperTRIBE have been widely applied to neuronal RBPs, revealing transcriptome-wide target sets that differ markedly from in vitro predictions and highlighting the importance of cellular context in RPI networks [94,154]. STAMP provides an orthogonal enzymatic tagging strategy and has been used to profile RNA targets of nuclear RBPs involved in transcriptional and post-transcriptional regulation [24,156]. APEX-seq further extends protein-centric analysis by labeling RNA populations within the spatial neighborhood of RBPs or subcellular landmarks, enabling discovery of localization-dependent RNA interaction patterns [24]. Crosslinking-based protein-centric approaches such as CLIP, iCLIP, and eCLIP remain the most stringent methods for identifying direct RNA–protein contacts at nucleotide resolution and have been essential for mapping binding sites of splicing factors and translational regulators [52,78]. These approaches are best suited when precise binding site information is required, although they provide limited insight into higher-order spatial organization.

When spatial organization or interaction dynamics are central to the biological question, imaging-based approaches offer indispensable complementary information. Fixed-cell imaging methods such as RNA FISH combined with immunofluorescence are appropriate for determining where RNA–protein associations occur within intact cellular architectures, as illustrated by visualization of β-actin mRNA–ZBP1 complexes during polarized cell migration and neuronal transport [219,220,221]. RNA FISH has also revealed co-enrichment of MALAT1 with SR-splicing factors within nuclear speckles, linking RNA localization to functional nuclear compartments [204,205,206,207]. RNA-PLA is particularly valuable when near-direct association must be demonstrated in situ, as it restricts signal generation to RNA–protein pairs within ~30–40 nm and has been used to validate both nuclear and viral RPIs [210]. Live-cell imaging approaches, including MS2/PP7-based RNA tagging and protein-centric tracking, extend these observations into the temporal domain, enabling direct visualization of RNP assembly, transport, and remodeling, such as the reversible binding of ZBP1 to β-actin mRNA during intracellular trafficking [219,220].

Across all methodological classes, careful attention must be paid to the spatial and temporal scale of detection. Direct molecular contacts occurring at sub-10 nm distances are most rigorously identified by UV crosslinking-based methods or FRET-based imaging, whereas proximity-based approaches capture association within broader nanometer-scale neighborhoods, and imaging-based methods often report RNP-level interactions spanning tens to hundreds of nanometers [209,218]. Recognizing these scale-dependent differences is essential for avoiding overinterpretation and for selecting complementary validation strategies.

Taken together, the examples highlighted here emphasize that effective RPI analysis depends on strategic integration of RNA-centric discovery, protein-centric specificity mapping, and imaging-based spatial and temporal validation. Such integrative experimental designs are increasingly necessary to move beyond static interaction catalogs toward mechanistic, context-aware models of RNA regulation, providing a natural transition to the challenges and limitations discussed in the following section.

## 4. Key Limitations in Studying RNA–Protein Interactions

Despite rapid methodological innovation, the study of RPIs remains constrained by trade-offs between resolution, sensitivity, physiological relevance, and scalability. A recurring challenge across platforms is the tension between biochemical specificity and preservation of native cellular context. Crosslinking-based methods such as CLIP and its derivatives rely on UV irradiation to stabilize RNA–protein contacts prior to purification, enabling nucleotide-level mapping of binding sites [78]. However, crosslink formation is inherently sequence- and protein-dependent, leading to uneven detection efficiency across transcripts and RBPs [23]. Reverse transcription truncation signatures used to infer contact sites can also be influenced by RNA secondary structure and local sequence context, introducing systematic bias [222].

Immunoprecipitation-based strategies, including RIP-seq and related workflows, are additionally dependent on antibody quality and epitope accessibility [20,68]. Variability in antibody specificity can result in nonspecific pulldown or inconsistent recovery across experiments [223]. Moreover, both native and crosslinked immunoprecipitation may capture indirect associations within large RNP assemblies rather than direct RNA binding, complicating mechanistic interpretation without orthogonal validation [78].

RNA-centric enrichment approaches such as hybridization-based pulldown methods (e.g., ChIRP, RAP-MS) face distinct limitations. Efficient capture depends on probe accessibility, which can be hindered by RNA structure, modification, or low transcript abundance [34,206]. Chemical crosslinkers preserve higher-order assemblies but frequently stabilize indirect interactions, broadening the apparent interactome beyond direct contacts [33]. Conversely, UV-based capture improves specificity but often yields low recovery efficiency [78]. CRISPR/Cas13–guided proximity labeling methods introduce programmable RNA targeting but remain sensitive to guide accessibility, off-target binding, and fusion protein expression levels [49,81]. Like other proximity systems, these approaches report molecular neighborhoods rather than atomic binding interfaces, requiring complementary validation to establish direct interaction sites.

Editing-based proximity recording systems including TRIBE and STAMP address crosslinking limitations by encoding RBP proximity directly into RNA sequence through nucleotide modifications [94,157]. While these methods operate in living cells and detect transient interactions, their readouts depend on local nucleotide availability and structural accessibility [153]. Editing frequency reflects proximity and dwell time rather than direct binding affinity, and an absence of editable nucleotides near a true binding site can generate false negatives [153,157]. Additionally, fusion protein expression levels must be carefully tuned to minimize background editing, particularly in tissues with endogenous RNA-editing activity [94].

Enzymatic proximity-labeling platforms such as APEX-seq provide high spatial resolution by biotinylating RNAs within defined subcellular microenvironments [24,163,164]. Because labeling occurs within a limited radius (~10–20 nm), enrichment reflects proximity rather than direct physical contact [79]. Although this property enables mapping of compartment-specific RNA–protein neighborhoods, it complicates interpretation when distinguishing direct binding from shared localization within dense RNP assemblies [24,42,163,164]. Furthermore, reactive oxygen species generated during APEX2 labeling can introduce cellular stress or nonspecific modifications if not carefully controlled.

Imaging-based approaches introduce a different spectrum of constraints. Fixed-cell techniques such as RNA-FISH combined with immunofluorescence or RNA–PLA provide valuable spatial context but are fundamentally limited by optical resolution (~200 nm for conventional microscopy), which exceeds the scale of direct molecular contact [22,204,205,206,207,210]. Super-resolution modalities (e.g., STORM, PALM, SIM) improve spatial precision to tens of nanometers but remain technically demanding, lower throughput, and dependent on complex image reconstruction pipelines [182,203].

Sensitivity is another critical limitation in imaging workflows. Low-abundance transcripts or weakly expressed RBPs may fall below detection thresholds, particularly in smFISH configurations where hybridization efficiency dictates signal recovery [182,203]. Background autofluorescence and nonspecific antibody binding can further reduce signal-to-noise ratios in complex tissues. While RNA–PLA enhances specificity by requiring dual recognition within ~30–40 nm, it still cannot discriminate direct binding from tight co-localization within multiprotein assemblies [22].

Multiplexing capacity also remains constrained. Although combinatorial RNA imaging platforms such as MERFISH and seqFISH achieve high RNA multiplexity, simultaneous detection of large numbers of proteins is limited by fluorophore availability, spectral overlap, and antibody compatibility [201,202]. As a result, most RNA–protein imaging experiments interrogate only a small number of molecules per assay, restricting their ability to capture multi-RBP regulatory networks in a single experiment. Live-cell RNA tagging systems (e.g., MS2/PP7) similarly face multiplexing limits due to the requirement for orthogonal stem-loop–coat protein pairs and compatible fluorescent channels [220,221].

Collectively, no single method simultaneously achieves nucleotide-level precision, high sensitivity for rare interactions, broad multiplexity, and minimal perturbation. Consequently, robust RPI analysis increasingly relies on integrating orthogonal techniques—combining, for example, CLIP-based site mapping with proximity labeling or imaging to reconcile binding specificity with spatial organization [24,78]. As methods evolve toward single-cell and spatially resolved frameworks, future progress will depend on balancing quantitative rigor with physiological fidelity to construct unified models of RNA–protein regulation in health and disease.

## 5. Conclusions

The field of RPI biology has progressed from reductionist biochemical assays to integrated, multi-scale frameworks that interrogate RNA–protein networks across molecular, cellular, and spatial dimensions. Crosslinking-based approaches such as CLIP and its derivatives established the first transcriptome-wide maps of RBP binding at near-nucleotide resolution, transforming our understanding of post-transcriptional regulation [23,78]. In parallel, RNA-centric pulldown strategies revealed how individual transcripts assemble dynamic RNP complexes that shape their stability, localization, and translation [31,32,33,34,35,36,37]. More recently, enzymatic proximity labeling and molecular recording systems—including TRIBE, STAMP, and APEX-seq—have enabled in vivo capture of RPI landscapes with temporal and spatial resolution that were previously unattainable [24,94,157]. Together, these advances have reframed RPIs not as isolated events, but as context-dependent regulatory networks embedded within cellular architecture.

One of the major conceptual shifts emerging from these technologies is the recognition that RPI networks are inherently dynamic and combinatorial. Many RBPs bind overlapping transcript pools, often in a position-dependent manner that dictates distinct functional outcomes, such as alternative splicing, translational repression, or RNA decay [9,52]. Increasingly, studies reveal that RBPs do not operate in isolation but form higher-order assemblies, frequently within phase-separated compartments such as stress granules or nuclear speckles [214,224]. The spatial organization of these assemblies influences transcript fate, linking RNA localization with processing efficiency and translational output [24,201]. This layered regulation underscores the importance of methods that preserve cellular context and resolve interactions within defined microenvironments.

Despite this progress, several limitations continue to constrain the interpretability and comparability of RPI datasets. Crosslinking-based methods provide precise binding maps but are influenced by UV crosslink bias and protein-specific crosslinking efficiencies [23,78]. Proximity-labeling approaches offer improved contextual fidelity but capture spatial neighborhoods rather than direct contact sites, necessitating orthogonal validation [24,157]. Editing-based recording systems depend on nucleotide accessibility and enzymatic preferences, potentially generating false negatives or quantitative ambiguity [94]. Imaging-based approaches deliver unparalleled spatial insight but remain limited by sensitivity, multiplexity, and resolution relative to molecular contact distances [200,225]. No single technique simultaneously achieves nucleotide-level precision, high sensitivity for rare interactions, quantitative affinity estimation, and minimal perturbation of native RNP organization.

Looking ahead, the future of RPI research lies in methodological integration and quantitative modeling. Combining orthogonal strategies such as pairing CLIP-based site mapping with proximity-labeling or single-cell transcriptomics can reconcile molecular specificity with physiological context [20,78,115]. Advances in enzyme engineering are expected to expand the mutational recording toolkit, enabling multi-channel proximity detection and temporally controlled labeling windows [94,163]. The incorporation of single-cell sequencing and spatial transcriptomics promises to resolve cell-state-specific RPI networks within heterogeneous tissues, an essential step toward understanding developmental transitions, immune responses, and tumor evolution [9,201].

Another frontier involves the integration of RPI data with complementary regulatory layers. Coupling RBP binding maps with ribosome profiling, chromatin conformation assays, and epitranscriptomic analyses will enable reconstruction of coordinated gene regulatory circuits spanning transcription, RNA processing, and translation [24,226]. Computational frameworks that model RBP occupancy as a probabilistic and dynamic variable rather than a static binary event will further refine predictive understanding of post-transcriptional regulation [227].

From a translational perspective, dysregulation of RBP–RNA networks has emerged as a recurrent theme in neurodegenerative disease, cancer, viral infection, and immune dysfunction [9,228]. High-resolution mapping of aberrant RPI landscapes can illuminate pathogenic mechanisms and identify therapeutic intervention points, including accessibility [229]. As RNA-targeted therapies mature, detailed knowledge of RBP binding logic will be critical for minimizing off-target effects and predicting network-wide consequences.

Ultimately, the trajectory of RPI research reflects a broader shift in molecular biology, from cataloging components to modeling systems. The next generation of technologies will likely focus on minimizing perturbation while maximizing resolution, enabling direct observation of RNP assembly and remodeling within living cells. By integrating biochemical precision with spatial and temporal context, the field is poised to move toward predictive, systems-level models of RNA–protein regulation that bridge molecular mechanism with organismal physiology.

## Figures and Tables

**Table 1:** Comparison of Hybridization-Based RNA Pulldown Platforms.

Technique	Crosslinking	Probe Design	Effective Proximity	Key Strengths	Main Limitations
iDRiP	UV (254 nm)	Short biotinylated probes	Direct RNA–protein contact (<1 nm)	Direct binding specificity	Low efficiency; UV bias [35,36]
ChIRP	Formaldehyde	Short biotinylated antisense DNA (~20 nt), tiled	Crosslinked RNP proximity (direct + indirect)	Simple design; widely used; good for chromatin RNAs	Indirect binders; background [31]
CHART	Formaldehyde	Short biotinylated probes guided by RNase H mapping	Similar to ChIRP; enriched for accessible regions	Improved specificity	Added optimization; lower throughput [32,33]
RAP-MS	Formaldehyde or UV	Long biotinylated probes (~90–120 nt)	Higher stringency; favors near-direct binders	High specificity; low background	High cost; large input [34]
MORPH-MS	Formaldehyde (or UV)	Unmodified antisense probes, universal biotin oligo	Direct, proximal interactions	Cost-effective; scalable	Not ideal for short RNAs [37]

**Table 2:** Comparison of Proximity Labeling-Based Protein Tagging Methods.

Approach	Enzymatic Platform	Targeting Strategy	Effective Proximity	Key Strengths	Main Limitations
HyPro-MS	APEX2	Antisense probe–mediated recruitment (fixed cells)	~20 nm	No genetic manipulation; applicable to primary/clinical samples	Fixed-cell only; proximity-based readout [44,45]
RNA-BioID	BirA	Endogenous RNA tagging via MS2/BoxB	~10 nm	Preserves native RNA regulation; transcript-specific	Genome editing required; low signal for rare RNAs [43]
BioID	BirA (mutant *E. coli* biotin ligase)	Fusion to RBPs or RNA-targeting modules	~10 nm	Captures weak and transient interactions; simple implementation	Long labeling times; background from diffusion or overexpression [39]
RaPID	BirA or BASU	λN–BoxB RNA tethering	~10 nm	Transcript-specific labeling in live cells	Requires RNA engineering; proximity ≠ binding [25]
APEX/APEX2	Engineered ascorbate peroxidase	Fusion to RBPs or RNA-targeting modules	~20 nm	High temporal resolution; suitable for dynamic assemblies	ROS-associated toxicity; requires careful optimization [41,42]

**Table 3:** Comparison of CRISPR-assisted proximity labeling methods.

Approach	Cas Platform	Labeling Chemistry	Effective Proximity	Key Strengths	Main Limitations
CARPID	dCas13	BASU/BirA	~10 nm	High sensitivity; endogenous RNA targeting; live-cell compatible	Proximity ≠ binding; guide accessibility critical [46]
CRUIS	dCas13	PUP-IT (uridylation tagging)	~10–20 nm	Orthogonal chemistry; reduced biotin-based background	Lower proteomic depth; complex workflow [48]
CBRPP	dCas13	Proximity biotinylation	~10–20 nm	Flexible; suitable for low-abundance and nuclear RNAs	Background labeling; requires stringent controls [47]
dCas13–APEX2	dCas13	APEX2 peroxidase	~20 nm	Rapid labeling; high temporal resolution	ROS-associated toxicity; narrow labeling window [42,49]
RPL-CLIP	dCas13	Proximity labeling + UV crosslinking	Direct binding (nucleotide-level)	Combines proximity mapping with binding specificity	Technically complex; low throughput [50]

**Table 4:** Comparison of Immunoprecipitation-Based Protein-Centric Methods.

Method	Crosslinker	Key Strengths	Main Limitations	Resolution
CLIP	UV (254 nm) crosslinking combined with immunoprecipitation and gel purification	First method to capture native RBP–RNA complexes in living cells; established the foundation for transcriptome-wide mapping	Low crosslinking efficiency; labor-intensive protocol; limited throughput	Provides site-specific interaction data; best for proof-of-principle studies and pioneering RBP–RNA mapping [20,78,115]
HITS-CLIP (CLIP-seq)	UV (254 nm) crosslinking of endogenous RPIs	Transcriptome-wide mapping; captures native interactions in vivo; widely validated	Low crosslinking efficiency; random crosslink positions; limited nucleotide resolution	Moderate resolution; suitable for global RBP–RNA landscape mapping [117,118]
PAR-CLIP	Photoreactive nucleoside analogs (e.g., 4-thiouridine, 6-thioguanosine) incorporated into RNA, then UV (365 nm) crosslinking	Higher crosslinking efficiency; diagnostic mutations (T→C) improve confidence; ideal for dynamic regulation studies	Requires nucleoside incorporation; limited use in primary tissues; analogs may perturb RNA metabolism	High confidence in binding sites; best for studying RNA turnover and dynamic RBP regulation [118,119]
iCLIP	UV (254 nm) crosslinking; reverse transcription, truncation marks, precise binding sites	Single-nucleotide resolution; identifies binding motifs and splicing regulation	Technically demanding; lower library complexity; potential variability	Single-nucleotide precision; best for mechanistic studies and motif discovery [23,122,123]
eCLIP	UV (254 nm) crosslinking with ENCODE-standardized pipeline and size-matched input controls	Streamlined workflow; reproducible and sensitive; lower input required; scalable for high-throughput	Moderate resolution; requires rigorous controls; may miss transient interactions	High-throughput and reproducible; best for systematic RBP profiling across conditions [15,52,124]

**Table 5:** Comparison of RNA Tagging Based Protein-Centric Methods.

Approach	Enzymatic Mechanism	Spatial Resolution	Temporal Resolution	Key Strengths	Main Limitations
TRIBE	ADAR catalytic domain fusion	~tens of nt around binding site	Moderate	In vivo recording; captures transient interactions; low input compatible	Dependent on accessible adenosines; editing bias; no nucleotide-level precision [94]
HyperTRIBE	Hyperactive ADAR (e.g., E488Q)	Similar to TRIBE	Improved sensitivity	Improved sensitivity over TRIBE	Increased background; expression tuning required [153,154]
STAMP	APOBEC1 cytidine deaminase fusion	~tens–hundreds of nt proximity window	Moderate	Captures targets lacking editable adenosines; single-cell adaptable	Dependent on cytidine context; proximity ≠ direct binding [157]
TRIBE-STAMP	Dual ADAR + APOBEC fusion system	Similar to editing radius	Moderate	Detects cooperative/competitive RBP binding on same RNA	Complex data analysis; higher background risk [161]
APEX-seq	APEX2 peroxidase proximity labeling	~10–20 nm proximity	High (seconds–minutes)	High spatial resolution; dynamic RNP remodeling detection	Labels proximity, not direct binding; ROS-induced artifacts [24,163]
PUP-Based RNA Tagging	Poly(U) polymerase (e.g., PUP-2) fusion	3′-end tagging; proximity-dependent	Moderate	Crosslink-free; quantitative tail length info	Sensitive to 3′-end accessibility; decay bias [169]
PIP-seq (adapted proximity systems)	Droplet-based + proximity chemistry	Single-cell resolution	High throughput	Enables single-cell RPI mapping	Requires complex library prep; computational filtering [173,174,175]

**Table 6:** Comparison of Fixed-Cell Imaging Methods.

Technique	Effective Proximity	Information Provided	RPIs Studied	Key Strengths	Main Limitations
FISH + IF	Diffraction-limited co-localization (~200–300 nm)	RNA localization and spatial association with RBPs	β-actin–ZBP1 (IGF2BP1); MALAT1–SRSF1/SRSF2; XIST–HNRNPU/SPEN	Single-molecule RNA detection; endogenous targets; compatible with tissues	Co-localization does not prove direct interaction [204,206]
MERFISH, seqFISH	Diffraction-limited; transcriptome-scale context	Spatial RNA programs and compartment-specific RBP recruitment	Nuclear lncRNA programs; cell-state-specific RNA signatures	High multiplexing; single-cell resolution	Complex probe design; indirect interaction inference [201,202]
RNA FISH + Super-Resolution Microscopy (STORM, PALM, SIM, SMLM)	~20–50 nm spatial resolution	Nanoscale organization of RNP assemblies	Nuclear speckle-associated lncRNAs; cytoplasmic RNP granules	Improved spatial precision; resolves dense assemblies	Technically demanding; interaction inferred by proximity [182,203]
RNA-PLA	~30–40 nm proximity threshold	Near-direct RNA–protein association in situ	MALAT1–lncRNA	High specificity; punctate readout; endogenous targets	Fixed cells only; cannot distinguish direct vs. indirect binding [22,210]
FRET-Based RNA–Protein Imaging (Fixed Cells)	<10 nm (direct-contact scale)	Direct RNA–protein proximity	Pre-mRNA-splicing factor interactions	Highest specificity for direct binding	Low throughput; requires engineered labeling [209]
ExM with RNA/Protein Labeling	Effective ~20–70 nm after expansion	RNA–protein spatial relationships in dense environments	Viral RNPs; RNA-rich compartments	Uses standard microscopes; scalable	Sample processing complexity [212,213]

**Table 7:** Comparison of Live-Cell Imaging Methods.

Technique	Effective Proximity	Information Provided	RPIs Studied	Key Strengths	Main Limitations
CRISPR/Cas13-Based RNA Imaging	Diffraction-limited (~200–300 nm)	Localization and dynamics of native RNAs	Nuclear lncRNAs; eRNAs	No RNA engineering; programmable	Signal-to-noise challenges; guide optimization [80,217]
Live-Cell RB-FRET	<10 nm (direct-contact scale)	Direct RNA–protein proximity	hnRNP H; its cognate RNA	High specificity for direct binding	Low throughput; engineered systems [218]
Live-Cell RNA Tagging (MS2, PP7, λN–BoxB)	Diffraction-limited (~200–300 nm); RNP-scale proximity	RNA localization, transport, and interaction dynamics	β-actin–ZBP1 (IGF2BP1); ASH1–She proteins	Single-RNA tracking; real-time dynamics; well-established	Requires RNA engineering; indirect interaction inference [185,219,220]
Protein-Centric Live-Cell Imaging)	Diffraction-limited; compartment-level proximity	RBP recruitment kinetics and residence times	β-actin–ZBP1	No RNA modification required; RBP dynamics	RNA association inferred indirectly [221]

## Data Availability

No new data were created or analyzed in this study. Data sharing is not applicable.

## Abbreviations

The following abbreviations are used in this manuscript:

RPI	RNA Protein Interaction
RBP	RNA Binding Protein
lncRNA	Long non-coding RNA
AD	Alzheimer’s Disease
ALS	Amyotrophic Lateral Sclerosis
Co-IP	Co-Immunoprecipitation
NGS	Next Generation Sequencing
CLIP	Crosslinking and Immunoprecipitation
RIP	RNA-Immunoprecipitation
RNA-PLA	RNA–Proximity Ligation Assay
iCLIP	Individual-Nucleotide Resolution Crosslinking and Immunoprecipitation
RAP-ID	RNA–Protein Interaction Detection (by Biotinylation)
LC-MS	Liquid chromatography–tandem mass spectrometry
RNP	Ribonucleoprotein
CRISPR	Clustered regularly interspaced short palindromic repeats
ChIRP	Chromatin Isolation by RNA Purification
CHART	Capture Hybridization Analysis of RNA Targets
RAP-MS	RNA Antisense Purification followed by Mass Spectrometry
iDRiP	identification of Direct RNA Interacting Proteins
MORPH	Multiple Oligo-Assisted RNA Pulldown via Hybridization
HyPro-MS	Hybridization Proximity followed by Mass Spectrometry
CARPID	CRISPR-assisted RNA–protein interaction detection
CBRPP	CRISPR-Based RNA Proximity Proteomics
CRUIS	CRISPR-based RNA-United Interacting System
RPL-CLIP	RNA proximity labeling combined with crosslinking immunoprecipitation
ChIRP-MS	Chromatin Isolation by RNA Purification followed by Mass Spectrometry
crRNA	CRISPR RNA
eRNA	Enhancer RNA
SELEX	systematic evolution of ligands by exponential enrichment
HTR-SELEX	High-throughput RNA SELEX
SEQRS	sequencing of RNA substrate specificity landscapes
LACE-seq	linear amplification of complementary DNA ends and sequencing
TRIBE	Targets of RNA-binding proteins Identified by Editing
RIP-seq	RNA immunoprecipitation followed by sequencing
PAR-CLIP	Photoactivatable Ribonucleoside-Enhanced CLIP
eCLIP	enhanced CLIP
ARTR-seq	Assay of reverse transcription-based RBP Binding Site Sequencing
RT&Tag	Reverse Transcribe and Tagment
STAMP	Surveying Targets by APOBEC-Mediated Profiling
HITS-CLIP	High-throughput sequencing-CLIP
OMM	outer mitochondrial membrane
ChromID	Chromatin Identification
PIP-seq	Particle-templated partition sequencing
FISH	fluorescence in situ hybridization
FRET	Förster resonance energy transfer
RNA FISH	RNA fluorescence in situ hybridization
smFISH	single-molecule RNA FISH
MERFISH	Multiplexed Error-Robust Fluorescence In Situ Hybridization
seqFISH	sequential Fluorescence In Situ Hybridization
RCA	rolling-circle amplification
STORM	Stochastic Optical Reconstruction Microscopy
PALM	Photoactivated Localization Microscopy
SMLM	Single-Molecule Localization Microscopy
SIM	Structured Illumination Microscopy
ExM	Expansion Microscopy
IPNR-PLA	Immunoprecipitation–Nucleic Acid Proximity Ligation Assay
APEX-Seq	Ascorbate Peroxidase–Sequencing
